# Stabilization of Non-Native Folds and Programmable Protein Gelation in Compositionally Designed Deep Eutectic Solvents

**DOI:** 10.1021/acsnano.4c01950

**Published:** 2024-07-01

**Authors:** Adrian Sanchez-Fernandez, Jia-Fei Poon, Anna Elizabeth Leung, Sylvain François Prévost, Cedric Dicko

**Affiliations:** † Center for Research in Biological Chemistry and Molecular Materials (CiQUS), Department of Chemical Engineering, Universidade de Santiago de Compostela, Santiago de Compostela 15705, Spain; ‡ European Spallation Source, Lund University, Lund SE-22100, Sweden; § Institut Laue-Langevin, DS/LSS, Grenoble 38000, France; ∥ Pure and Applied Biochemistry, Department of Chemistry, Lund University, Lund SE-22100, Sweden; ⊥ Lund Institute of Advanced Neutron and X-ray Science, Lund SE-22370, Sweden

**Keywords:** deep eutectic solvent, protein conformation, folding intermediates, supramolecular entanglement, protein eutectogel

## Abstract

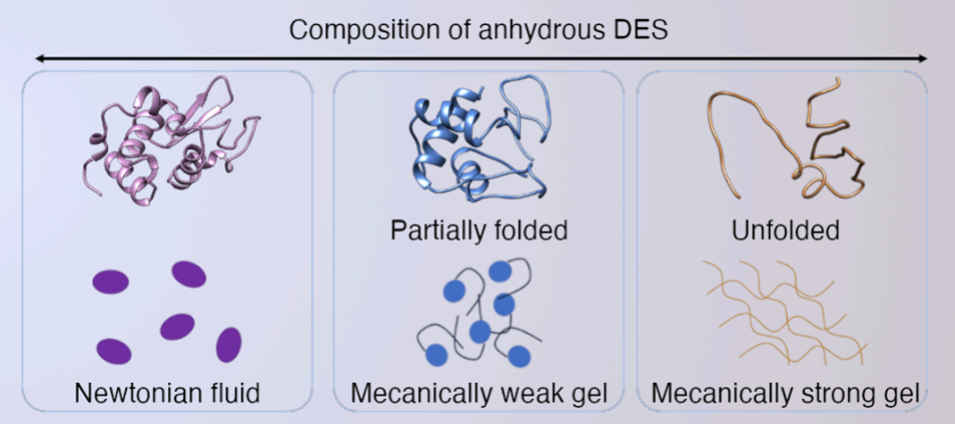

Proteins are adjustable units from which biomaterials with designed properties can be developed. However, non-native folded states with controlled topologies are hardly accessible in aqueous environments, limiting their prospects as building blocks. Here, we demonstrate the ability of a series of anhydrous deep eutectic solvents (DESs) to precisely control the conformational landscape of proteins. We reveal that systematic variations in the chemical composition of binary and ternary DESs dictate the stabilization of a wide range of conformations, that is, compact globular folds, intermediate folding states, or unfolded chains, as well as controlling their collective behavior. Besides, different conformational states can be visited by simply adjusting the composition of ternary DESs, allowing for the refolding of unfolded states and vice versa. Notably, we show that these intermediates can trigger the formation of supramolecular gels, also known as eutectogels, where their mechanical properties correlate to the folding state of the protein. Given the inherent vulnerability of proteins outside the native fold in aqueous environments, our findings highlight DESs as tailorable solvents capable of stabilizing various non-native conformations on demand through solvent design.

Since the emergence of “Levinthal’s paradox”, protein folding is described by the overarching model of the folding funnel. In a nutshell, proteins travel through multiple energetic routes during folding until they reach the low-energy level associated with the compact native state.^1−3^ As the protein folds, the intermediate states correspond to transient conformations that progressively evolve and change. These ephemeral intermediates, which constitute partially formed secondary and tertiary structures within shallow energy wells,^4^ are generally challenging to isolate in an aqueous milieu.^5^ While nature employs molecular chaperones to protect folding intermediates against degradation,^6^ providing an artificial environment for partial folds in the absence of chaperones remains challenging. However, gaining access to these non-native conformations holds the potential to deepen our understanding of folding mechanisms, protein function, and biomaterial development.^7−9^

By introducing nonaqueous enzymology,^10,11^ organic solvents have expanded the possibilities of protein-based technologies beyond aqueous environments. In this paradigm, the properties of the solvent could be selected to improve the stability and function of proteins. For example, substrate and product selectivity can be tailored by changing the solvent.^12^ However, denaturation often ensues upon incorporation of proteins in organic solvents. Thus, incorporation in early studies was achieved using suspended powders and immobilized enzymes, where the biomolecule resides in a highly stable, kinetically arrested state.^12^ Later, the realm of nonaqueous enzymology was further expanded by introducing ionic liquids (ILs) as “designer” media for protein stabilization and function. The task-specific character of ILs potentially allows the development of solvents where protein performance and stability can be finely tuned through changes in the solvent properties.^9,13,14^ The vast landscape of possible solvent–protein interactions involving systematic permutations in IL constituents can open a wide range of conformational changes in a protein,^15^ where the effect of solvent-on-protein is as essential as that of protein-on-solvent.^16,17^ However, the harsh character of many ILs could hinder applications involving labile biomolecules and health-related applications.^13^

At the crossroads of protein solubilization media and nonaqueous solvents, DESs have emerged as suitable environments due to their mild character, thermal and chemical stability, and potential biocompatibility.^18,19^ These solvents are formed by mixing a hydrogen bond donor (HBD) and an acceptor (HBA) at a specific ratio, resulting in a eutectic mixture characterized by a depression in the melting point that allows the system to remain liquid at room temperature.^20−22^ DESs share the tunable character of ILs, that is, changes in the constituents of the solvent lead to controlled variations in the physicochemical properties of the solvent, for example, polarity, hydrogen potential, and hydrophobicity.^19,23−25^ The wide range of possible protein–DES interactions, such as tailored hydrogen bonds, salt bridges, or solvophobic effects, lets us envision the possibility of developing “designer” solvents to solubilize amino acid sequences, improve protein stability, and modulate their function.^26,27^ As such, protein solubilization into DESs finds prospective applications in designed enzymatic catalysis,^28^ protein preservation,^29^ selective extractions,^30^ development of functional biomaterials,^31^ and improved formulation technologies.^18^ This popularity is reflected in the rapid increase in the number of investigations in protein behavior in DESs,^26^ with an ever-expanded understanding of the complex process of protein solubilization in these solvents; for example, the ability of DESs to support protein folding into defined conformations,^29,32,33^ the formation of a solvation matrix based on an extensive hydrogen bond network,^34,35^ the existence of specific protein–solvent interactions that affect protein conformation,^36,37^ and the enhancement of protein stability.^38,39^

The exploration of technologies and materials involving kinetically trapped unfolded or partially folded states can be used in highly regulated protein assemblies (e.g., gels) and programmed molecular recognition (e.g., ligand promiscuity).^9,40,41^ As tailorable and mild environments, DESs could potentially access and stabilize non-native conformations simply by varying the solvent’s inherent properties, thus expanding the dimensions of protein-based systems beyond what we can access with traditional solvents. However, a general framework of the biophysical behavior of proteins as a function of DES components has yet to be found. In response to this challenge, we have investigated the ability of neat (anhydrous) binary and ternary DESs to control the conformation of two model proteins, lysozyme (Lyz) and bovine serum albumin (BSA). Preliminary investigations have shown the ability of neat 1:2 choline chloride:glycerol to solubilize these proteins.^38^ Besides, these two proteins have been extensively characterized in aqueous environments, thus constituting a valuable baseline for our results. The DESs used in our study were selected due to their well-known properties (Figure 1a): 1:2 choline chloride:glycerol (1:2 ChCl:Glyc),^42^ 1:2 choline acetate:glycerol (1:2 ChAcO:Glyc),^43^ 1:2 choline chloride:acetic acid (1:2 ChCl:AcOH),^44^ and 1:2 choline chloride:urea (1:2 ChCl:Urea).^20^ Also, the possibility of using a constant 1:2 halide salt-to-hydrogen bond donor ratio allows us to discern changes in the protein when introducing systematic variations in the solvent constituents and the direct preparation of ternary DESs (Figure 1b). Here, we present an amenable platform based on compositionally designed DESs to access a broad conformational landscape, where systematic variations in the solvent composition allow for the stabilization of a wide range of folding states, from globular folds to a range of partially folded and unfolded conformations. In addition, DESs modulate the collective behavior in a colloidally stable state. Those non-native folds can yield the formation of protein (eutecto)gels, where their mechanical properties are defined by the folding state of the protein, ultimately controlled by the solvent composition. As such, these results, summarized in Figure 1, provide the grounds to stabilize folding intermediates, from which macroscopic responses can be triggered.

**Figure 1:**
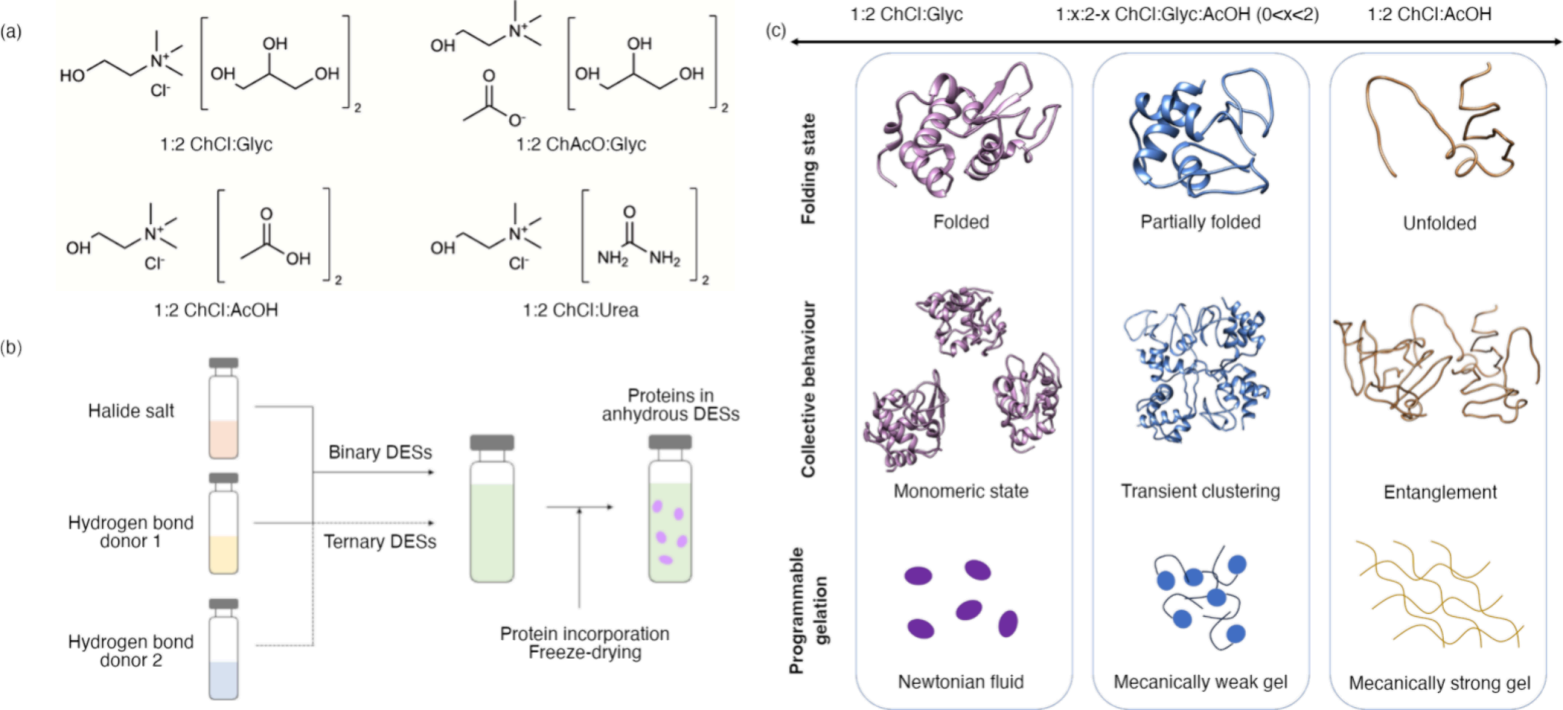
Overview of the procedure to modulate protein conformation using compositionally designed DESs: (a) chemical structures of the compositionally varied binary DESs. (b) Schematic of the preparation of binary and ternary DESs and the protocol for protein incorporation yielding anhydrous systems. (c) Proposed conformational states stabilized with varying the composition of the ternary DESs, which also results in differences in the collective behavior of the protein; the topological interactions between proteins define the mechanical response of the system in a programmable manner, where the entanglement of partially unfolded and unfolded intermediates can be used to trigger gelation with varied mechanical properties.

## Results and Discussion

### Conformational Boundaries in Binary DESs

Our initial studies were oriented toward the conformational aspects of the two model proteins in binary DESs with systematically varied composition. The characteristic behavior of the two proteins in aqueous buffer (10 mM, pH 7 sodium phosphate buffer, herein referred to as the native state) was used as the baseline comparison. UV–vis absorption, intrinsic Trp fluorescence spectroscopy (λ_ex_ = 295 nm), and far-UV circular dichroism (CD) were employed to investigate the solvation mechanism and secondary structure of the two proteins in the different DESs. Small-angle neutron scattering (SANS) was used to investigate the tertiary and quaternary structures of the proteins as a function of solvent composition. Note that the SANS investigation requires the use of isotopically labeled DES, and the deuteration of the DESs has been previously shown to cause negligible changes in the behavior of proteins compared to the protiated analogues.^33,38^ SANS data were analyzed using the indirect Fourier transform (IFT) method to obtain the pair-distance distribution functions (*p*(*r*)) of the proteins.^45,46^ Further details on the data analysis and a complete record of the results are presented in the Supporting Information.

**Figure 2:**
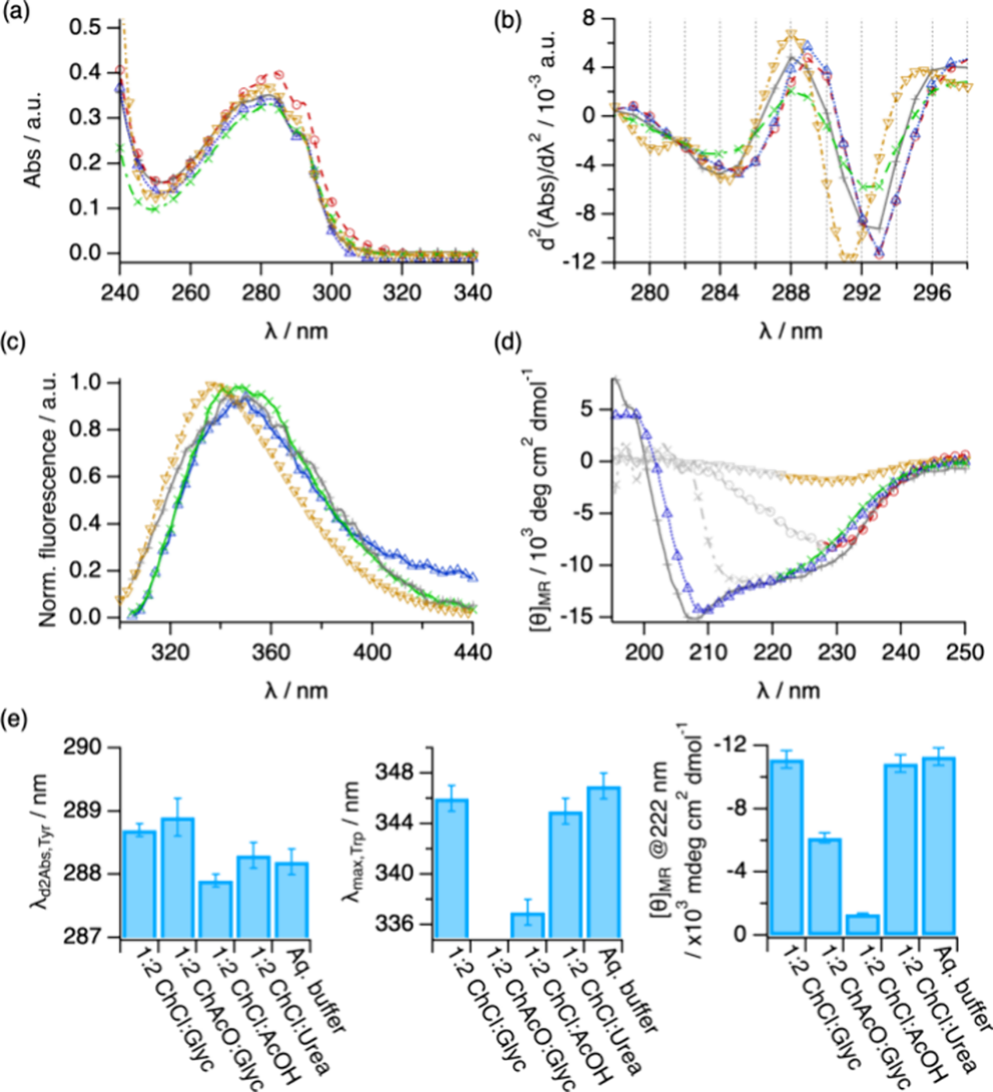
Results from the characterization of Lyz in aqueous buffer (grey plus symbol), 1:2 ChAcO:Glyc (red circle open), 1:2 ChCl:Glyc (blue triangle up open), 1:2 ChCl:Urea (green cross mark), and 1:2 ChCl:AcOH (brown triangle down open): (a) UV–vis absorption, (b) second derivative UV–vis, (c) normalized fluorescence emission, and (d) far-UV CD spectra. Data in panel (d) obscured by solvent absorbance are presented in light gray. (e) Parameters derived from the analysis of the data for Lyz in different DESs and aqueous buffer: λ_d2,Tyr_, Tyr peak position obtained from the peak analysis of the second-derivative UV–vis spectra, λ_max,Trp_, position of the emission maximum for the Trp peak, and [θ]_MR_, mean residue ellipticity at 222 nm. The error bars represent the standard deviations from the averaged values. Where not seen, error bars are within the markers.

Upon Lyz incorporation, the samples were visually inspected and we observed that the protein remained solubilized in all DESs. The UV–vis data show the characteristic absorption peak centered at about 280 nm associated with the chromophores’ absorbance and concentration (Figure 2a). The spectra almost overlap for all of the DESs and aqueous buffer, thus confirming that the protein was effectively incorporated into all solvents. To extract detailed information on the changes in the environment of the protein’s Tyr residue, second-derivative UV–vis spectra (d^2^(Abs)/dλ^2^) were calculated and analyzed (Figure 2b).^47^ For Lyz, a subtle bathochromic shift is observed for λ_d2,Tyr_ when solvated in 1:2 ChCl:Glyc, 1:2 ChAcO:Glyc, and 1:2 ChCl:Urea, whereas Lyz in 1:2 ChCl:AcOH displays a hypsochromic shift compared to the native behavior (Figure 2a,b,e). The Trp emission results (λ_max, Trp_) confirm these observations (Figure 2c,e), with minor changes (within error) in the emission maximum for the former DESs and a more significant hypsochromic shift in the case of the acid–based DES. It should be noted that fluorescence data could not be acquired in 1:2 ChAcO:Glyc due to the absorption from the solvent in the emission window of Trp. To determine the origin of these changes in the chromophore environment, the secondary structure of Lyz was investigated by far-UV CD.^48^ Since 1:2 ChAcO:Glyc, 1:2 ChCl:Urea, and 1:2 ChCl:AcOH obscure the acquisition of CD data below 215 nm despite the short path length of the cuvette, the mean residue ellipticity ([θ]_MRE_) at 222 nm was used to quantify the amount of ordered secondary structure of the protein in DESs. The spectra show clear differences between the systems investigated here. Lyz retains a near-native secondary structure when dissolved in 1:2 ChCl:Glyc, with similar spectral features to those in the aqueous buffer (Figure 2d,e). Replacing the hydrogen bond donor with acetic acid leads to a complete loss of the far-UV CD signal. This vanished signal is attributed to the disruption of the ordered secondary structure motifs and the concomitant loss of the chiral environment of the protein backbone. The secondary structure in 1:2 ChCl:Urea and in 1:2 ChAcO:Glyc seems somewhat retained, although the strong adsorption from the solvent does not allow us to define the region of the negative peak at 209 nm.

**Figure 3:**
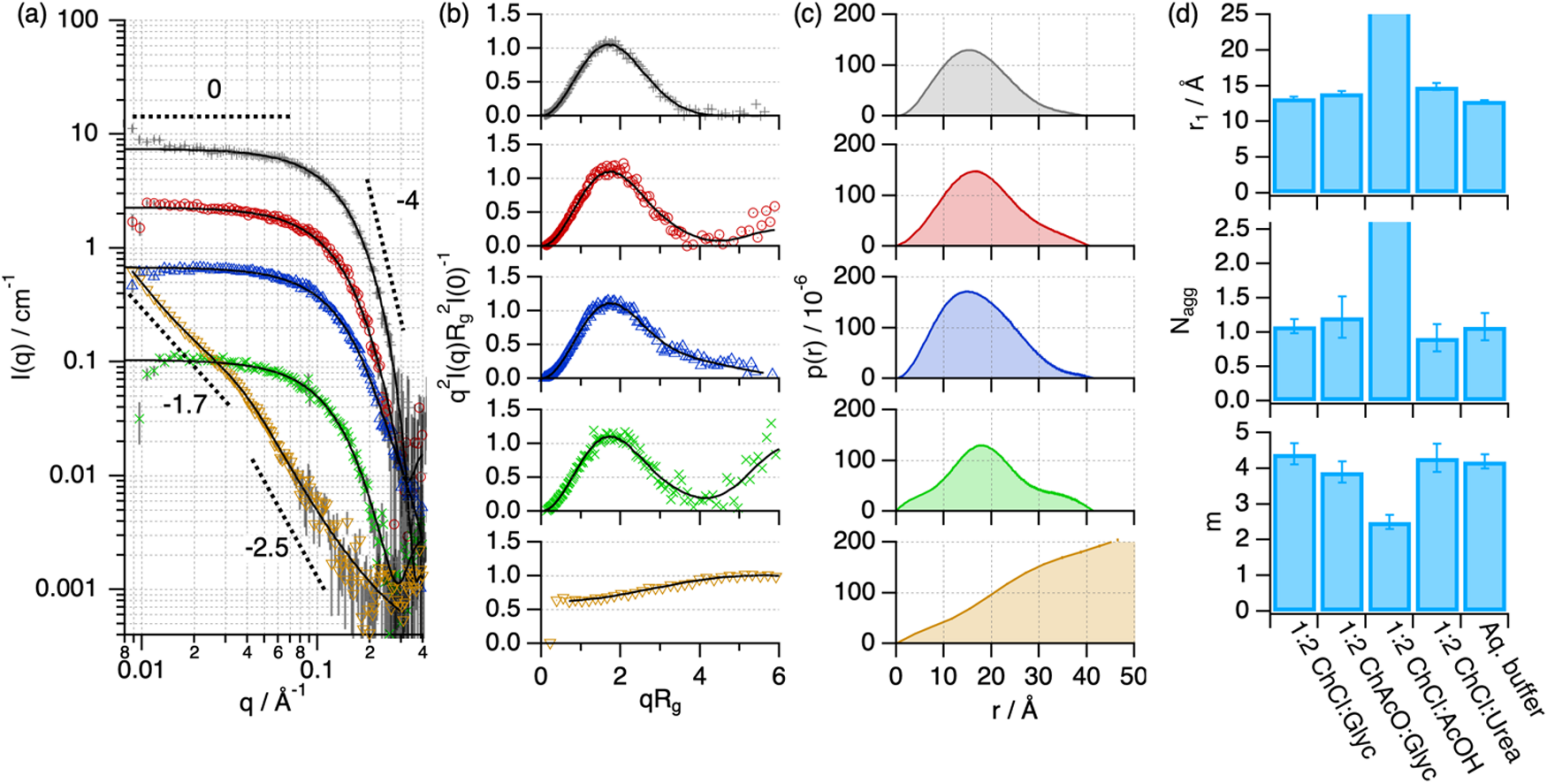
SANS data and best fits of Lyz in aqueous buffer (grey plus symbol), 1:2 ChAcO:Glyc (red circle open), 1:2 ChCl:Glyc (blue triangle up open), 1:2 ChCl:Urea (green cross mark), and 1:2 ChCl:AcOH (brown triangle down open). The SANS models obtained from IFT analysis (folded states) or generalized Porod analysis (unfolded states) are presented as black solid lines. The dashed lines represent representative Porod slopes. (a) Data and models have been offset for clarity by n*2. (b) The normalized Kratky plot of the SANS data and models and (c) the *p*(*r*) of the protein in the different solvents. (d) Parameters derived from the structural analysis of the protein: *r*_1_, the apparent size of the protein monomer parametrized as the position of the first peak in the *p*(*r*), *N*_agg_, the average number of protein units in a self-association equilibrium, and *m*, negative Porod slope at high *q*. The error bars represent the standard deviations from the averaged values. Where not seen, error bars are within the markers.

The SANS characterization provides detailed information on the overall conformation of the protein, that is, folding, shape, and size.^46^ In particular, the Kratky representation of the scattering data enables a direct qualitative assessment of the protein folding state, where a bell-shaped peak at q*R*_g_ < 1.7 and a sharp decay at q*R*_g_ > 1.7 correspond to a well-folded globular protein.^49^ However, a plateau at q*R*_g_ > 1.7 relates to unfolded states. The SANS characterization of Lyz in DESs shows that the protein in 1:2 ChCl:Glyc, 1:2 ChAcO:Glyc, and 1:2 ChCl:Urea retains a globular conformation similar to that in the native state, as seen in the Kratky plots (Figure 3a,b).^50,51^ The calculated pair-distance distribution functions (Figure 3c), which represent a histogram of distances within the scatterer,^46^ confirm the globularity of the protein in these systems. Interestingly, only a slight increase in size (ca. 4%) is observed when comparing the characteristic dimension of the protein monomer, r_1_, in these DESs to that in the native state, relating to minimal changes in the folding (Figure 3c). Also, no changes occur in the self-association of the protein, thus predominantly remaining as a monomer. In stark contrast, Lyz is unfolded in 1:2 ChCl:AcOH, confirmed by the generalized Porod analysis with a slope at high *q* of 2.5 (Figure 3d, e), which indicates a significant degree of interfacial disorder.^50^ In this unfolded state, Lyz adopts a random chain configuration, which correlates with the loss of the native secondary structure (vide supra). Furthermore, the increased scattering signal at low *q* is attributed to the supramolecular association of the unfolded chains, where the slope at low q of the SANS data (*n* ≈ 5/3) suggests the formation of a clustered network of swollen, polymer-like chains with large aggregation numbers (>12).

**Figure 4:**
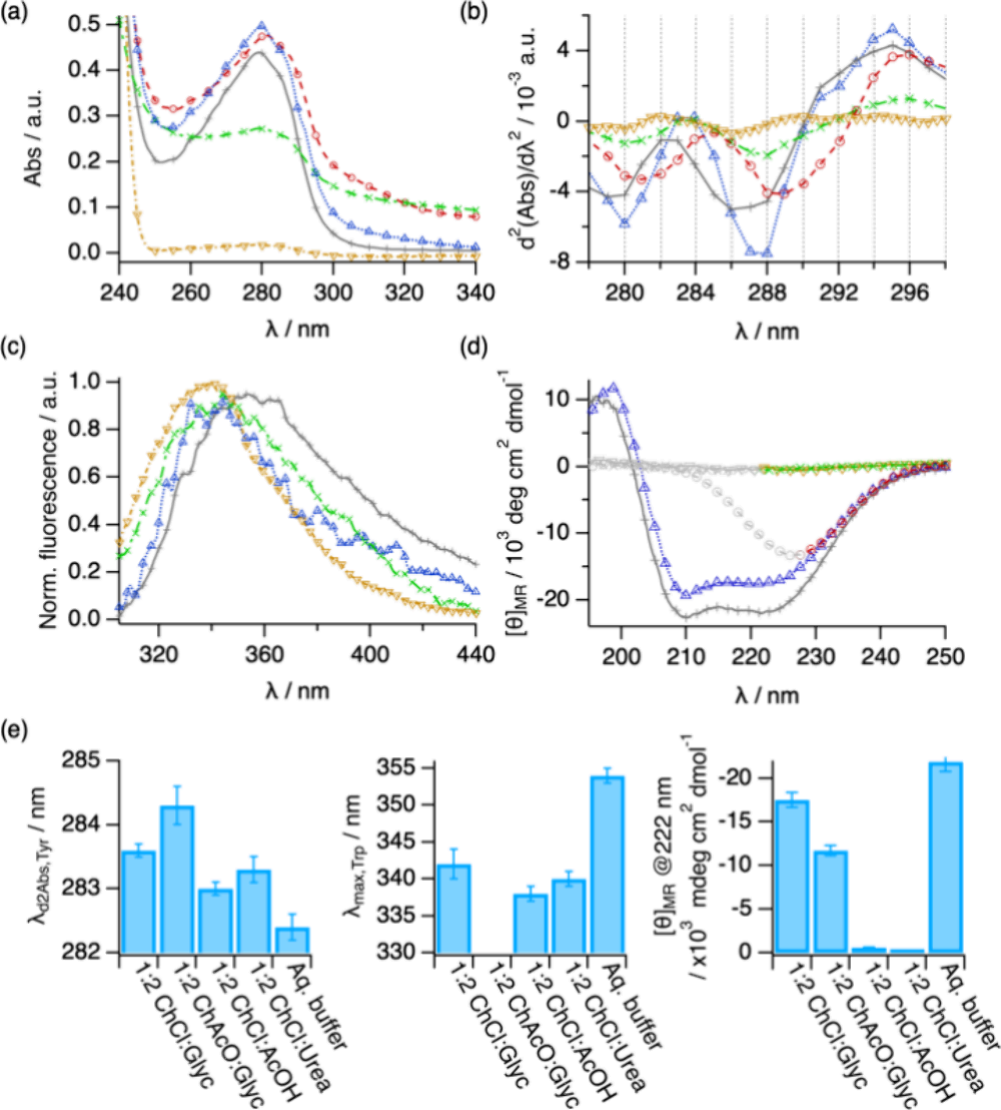
Results from the characterization of BSA in aqueous buffer (grey cross mark), 1:2 ChAcO:Glyc (red circle open), 1:2 ChCl:Glyc (blue triangle up open), 1:2 ChCl:Urea (green cross mark), and 1:2 ChCl:AcOH (brown triangle down open): (a) UV–vis absorption, (b) second derivative UV–vis, (c) normalized fluorescence emission, and (d) far-UV CD spectra. Data in panel (d) obscured by solvent absorbance are presented in light gray. (e) Parameters derived from the analysis of the data for Lyz in different DESs and aqueous buffer: λ_d2,Tyr_, Tyr peak position obtained from the peak analysis of the second-derivative UV–vis spectra, λ_max,Trp_, position of the emission maximum for the Trp peak, and [θ]_MR_, mean residue ellipticity at 222 nm. The error bars represent the standard deviations from the averaged values. Where not seen, error bars are within the markers.

In contrast, BSA was more susceptible to changes in the solvent composition than Lyz. BSA could be solubilized in only 1:2 ChCl:Glyc and 1:2 ChAcO:Glyc, but large aggregates formed when we attempted to incorporate it into 1:2 ChCl:Urea and 1:2 ChCl:AcOH. This behavior is also reflected in the UV–vis data. As protein solutions in the different DESs and aqueous buffer were prepared at nearly identical concentrations (ca. 100 μM), the much weaker absorbed intensity in 1:2 ChCl:Urea and 1:2 ChCl:AcOH was attributed to a considerably lower amount of solubilized protein (Figure 4a). The shifts in the characteristic spectral features become more pronounced than in the case of Lyz, thus suggesting that the chromophores further deviate from their native environment when solubilized in 1:2 ChCl:Glyc and 1:2 ChAcO:Glyc (Figure 4b,c). These changes are likely attributed to variations in the solution structure of BSA, as the CD spectrum of BSA in 1:2 ChCl:Glyc shows a partial loss of the negative signals at 209 and 222 nm compared to that in aqueous buffer (Figure 4d), aligning with variations in the local folding of the protein compared to the native state, as previously reported.^38^ The overlay between the partial signal of BSA in 1:2 ChAcO:Glyc and the spectrum in 1:2 ChCl:Glyc suggests that the protein retains, at least partially, the secondary structure topology when the anion of the solvent. However, a detailed assessment could not be performed due to the obscured signal at short wavelengths. In contrast, the CD signal of BSA in 1:2 ChCl:Urea and 1:2 ChCl:AcOH completely vanished, likely due to the low protein concentration upon protein precipitation.

**Figure 5:**
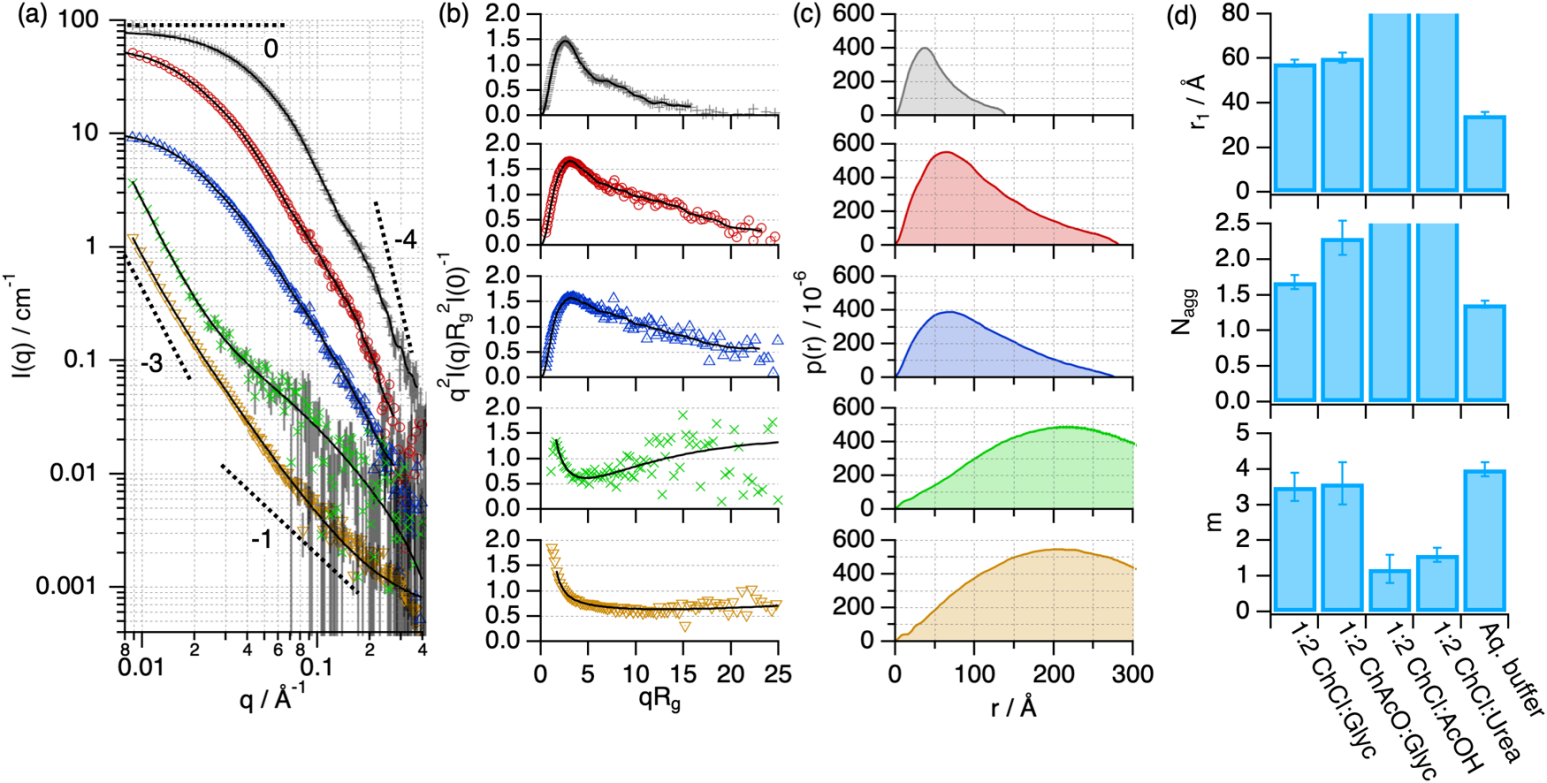
SANS data and best fits of BSA in aqueous buffer (grey plus symbol), 1:2 ChAcO:Glyc (red circle open), 1:2 ChCl:Glyc (blue triangle up open), 1:2 ChCl:Urea (green cross mark), and 1:2 ChCl:AcOH (brown triangle down open). The SANS models obtained from the IFT analysis (folded states) or generalized Porod analysis (unfolded states) are presented as black solid lines. The dashed lines represent representative Porod slopes. (a) Data and models have been offset for clarity by n*2. (b) The normalized Kratky plot of the SANS data and models and (c) the *p*(*r*) of the protein in the different solvents. (d) Parameters derived from the structural analysis of the protein: *r*_1_, the apparent size of the protein monomer parametrized as the position of the first peak in the *p*(*r*), *N*_agg_, the average number of protein units in a self-association equilibrium, and *m*, negative Porod slope at high *q*. The error bars represent the standard deviations from the averaged values. Where not seen, error bars are within the markers.

The results from the SANS characterization confirmed that these differences in the internal topology of BSA translate into changes in its overall conformation (Figure 5a). In the native state, the conformation of BSA in solution can be described as a mixture of folded (globular) monomers and dimers in a dynamic equilibrium.^52^ The normalized Kratky plots from BSA in 1:2 ChCl:Glyc and 1:2 ChAcO:Glyc show a bell-shaped feature at low *qR*_g_ and a less pronounced decay at *qR*_g_ > 5 (Figure 5b). Also, the nominal size attributed to the tertiary structure of the protein (*r*_1_) increases when BSA is solvated in 1:2 ChCl:Glyc and 1:2 ChAcO:Glyc compared to the native state (Figure 5c,d). These larger values and the shallow slope of the Kratky plot at high *qR*_g_ confirm that the BSA monomer is partially folded in these DESs when compared with the native state. The calculated *p*(*r*) of BSA shows an increased maximum dimension (*D*_max_), that is, the largest dimension observed for the scatterer in solution. In the glycerol-based DESs *D*_max_ doubles the value of the protein in aqueous buffer (ca. 140 vs 280 Å). Also, the BSA self-association, parametrized as the aggregation number (*N*_agg_), increases in the two glycerol-based DESs compared to the native state, suggesting that the native monomer–dimer equilibrium shifts toward the formation of larger oligomers, for example, a monomer–dimer-tetramer equilibrium.^38^ This change in the self-association can be attributed to a decrease in the screening length in DES compared to water, favoring specific protein–protein attraction and the formation of transient oligomers in 1:2 ChCl:Glyc and 1:2 ChAcO:Glyc.^53,54^ In 1:2 ChCl:Urea and 1:2 ChCl:AcOH, the weaker scattered intensity at high *q* confirms the poor solubility of BSA in these DESs, correlating with the aggregation previously observed. Regarding the solution structure of the remanent protein, the high-*q* Porod slope value (*m* ≈ 1) can be attributed to wholly unfolded protein domains, effectively behaving as a 1D polymer with a collapsed structure (*n* ≈ 3). These results confirmed that BSA unfolds in 1:2 ChCl:Urea and 1:2 ChCl:AcOH, and that the unfolding leads to rapid, nonspecific aggregation.

These observation agree with previous investigations that have reported that glycerol-based DESs (often 1:2 ChCl:Glyc) can solubilize proteins such as α-chymotrypsin, Lys, BSA, and subtilisin.^32,33,55,56^ However, our results demonstrate that the conformational state depends on the protein itself and on the properties of the solvent, where changes in the hydrogen bond donor have the most significant effect among the solvents investigated here, and the replacement of the anion subtly influences protein behavior. The anhydrous DESs 1:2 ChCl:Glyc, 1:2 ChAcO:Glyc, and 1:2 ChCl:Urea allow Lyz folding. However, it is observed that there is a subtle internal redistribution of the protein residues compared to the native aqueous behavior. This is supported by recent investigations, where site-specific protein–DES interactions (e.g., Trp-Ch^+^) cause these changes while preserving the overall globularity.^35,36^ In contrast, the acidic DES leads to the denaturation of the Lyz secondary structure and protein unfolding. A similar conformational transition has been observed in aqueous solutions of Lyz containing acetic acid, where the weak acid alters the native intraprotein interactions and hinders its folding.^57^ The solubilization of BSA in 1:2 ChCl:Glyc and 1:2 ChAcO:Glyc leads to the stabilization of conformational intermediates that differ in monomer fold and self-association to the native state. Again, these effects must be driven by interactions with the solvent, which causes a partial melting of the secondary structure motifs and the expansion of the protein structure.^37,38^ In 1:2 ChCl:Urea and 1:2 ChCl:AcOH, severe BSA denaturation is observed, leading to rapid protein aggregation. In summary, our hypothesis that the composition of DESs can prompt the stabilization of various folding states comes to realization.

### Designing Ternary DESs to Control Protein Folding

From the unending number of precursors for DESs, an immense range of potential properties ensues, yet predicting the suitable composition for a particular application remains challenging.^19^ As we sought to dive into the conformational landscape of proteins, instead of finding the required solvent properties within a (virtually) infinite number of possibilities, we turned to the simple approach of using ternary DESs. These hybrid systems consist of mixtures of two binary DESs with, for instance, the same halide salt (choline chloride) and a mixture of HBDs (glycerol and acetic acid), generally leading to solvents with intermediate properties to those of the constituent parts.^58^ Lyz was selected as model protein for these investigations since it shows high stability and low aggregation tendency in DESs,^29^ and thus was found more resilient than BSA. In addition, it has been previously reported that Lyz conformation can be modulated by cosolvency effects.^59^ Thus, we explored the conformation of Lyz in 1:*x*:2–*x* ChCl:Glyc:AcOH, with *x* = 1.9, 1.8, 1.75, 1.7, 1.65, 1.6, 1.5, 1.0, and 0.5. We selected these ternary DESs as the protein sits at the conformational boundaries in its binary counterparts: Lyz retains a native-like fold in 1:2 ChCl:Glyc, while it entirely unfolds in 1:2 ChCl:AcOH (vide supra).

**Figure 6:**
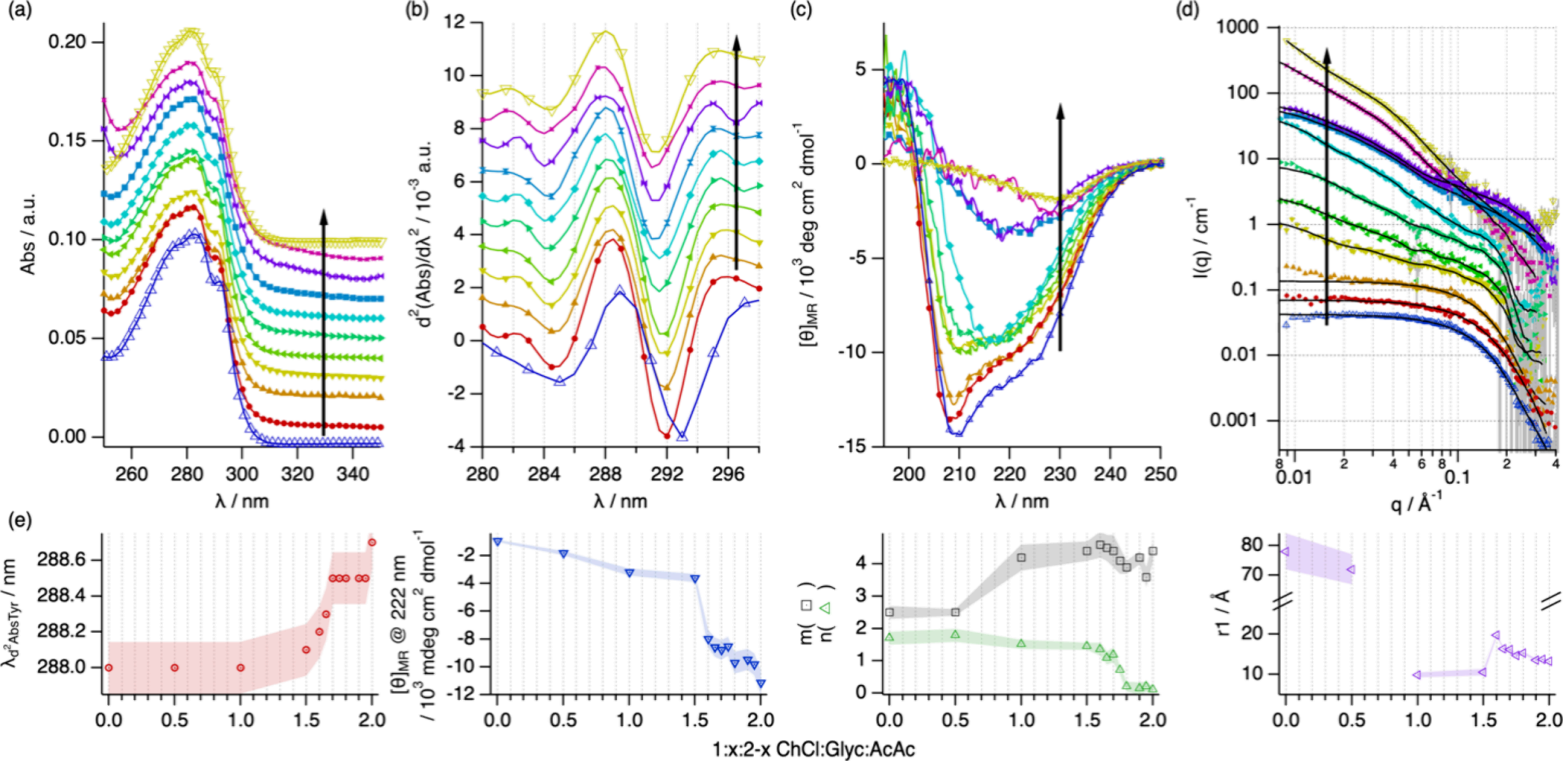
(a) UV–vis spectra, (b) second-derivative UV–vis spectra, (c) far-UV CD spectra, and (d) SANS data and best fits for Lyz in 1:*x*:2–*x* ChCl:Glyc:AcOH at 1:2:0 (blue triangle up open), 1:1.9:0.1 (red circle solid), 1:1.8:0.2 (orange triangle up solid), 1:1.75:0.25 (yellow triangle down solid), 1:1.7:0.3 (yellow-green triangle left-pointing solid), 1:1.65:0.35 (green triangle right-pointing solid), 1:1.6:0.4 (cyan tilted square solid), 1:1.5:0.5 (blue box solid), 1:1:1 (violet star solid), 1:0.5:1.5 (purple cross mark), and 1:0:2 (brown triangle down open). Data have been offset for clarity by (a) + n*0 01, (b) + n*0 001, and (d) n*2. The ratio of acetic acid in the DESs increases in the direction of the arrows. The main results derived from the analysis of the spectroscopy and scattering data (e): position of the Tyrosine peak (λ_d2AbsTyr_), mean residue ellipticity at 222 nm, Porod slopes at high (*m*) and low (*n*) *q*, and the apparent size of the protein monomer (*r*_1_). Where not seen, error bars are within the markers.

While Lyz remains stable in all the ternary DESs, our characterization reveals a nonlinear change in its behavior with varying DES composition (Figure 6). The spectral features attributed to the folded state observed in 1:2 ChCl:Glyc transition toward the unfolded state with increasing the AcOH content in the solvent. In the range 1.8 < *x* < 1.9, only subtle differences appear in protein spectral behavior, associated with a near-native secondary structure and a globular fold. Lyz in the ternary DESs with compositions 1.6 < *x* < 1.8 shows the characteristics of a folding intermediate, where subtle changes in the spectral parameters possibly relate to the exposure of the protein chromophores to the DES (Figure 6a,b). CD and SANS results reveal a partially preserved secondary structure and a partial unfolding of the overall Lyz structure. Interestingly, a gradual increase in the scattered intensity at low *q* appears in the SANS data, associated with the emergence of attractive protein–protein interactions and supramolecular clustering. The main transition in the spectral features occurs between 1 < *x* < 1.5, where most of the ordered secondary structure vanishes (Figure 6c). At this stage, a significant decrease occurs in the characteristic size of the protein monomer (Figure 6d), where *r*_1_ reaches the lowest values. Considering the loss of the defined secondary structure, this structural change is related to the complete unfolding of the tertiary structure of Lyz. In addition, the increased scattered intensity at low *q* reveals a change in protein self-association, which can be attributed to the swelling of these unfolded domains. At *x* = 0.5, the characteristic size of the monomer increases beyond again, confirming a transition to a conformational state where the protein domains collapse and enter a regime defined by the entanglement of polypeptide chains without helical secondary structures. Therefore, variations in the composition of the DESs can be used to control the structure and self-association of Lyz (Figure 6e), ranging between discrete globular folds (1.8 < *x* < 2), expanded molten globules (1.6 < *x* < 1.7), swollen polypeptide coil (1 < *x* < 1.5), and crumpled entangled chains (0 < *x* < 0.5).

**Figure 7:**
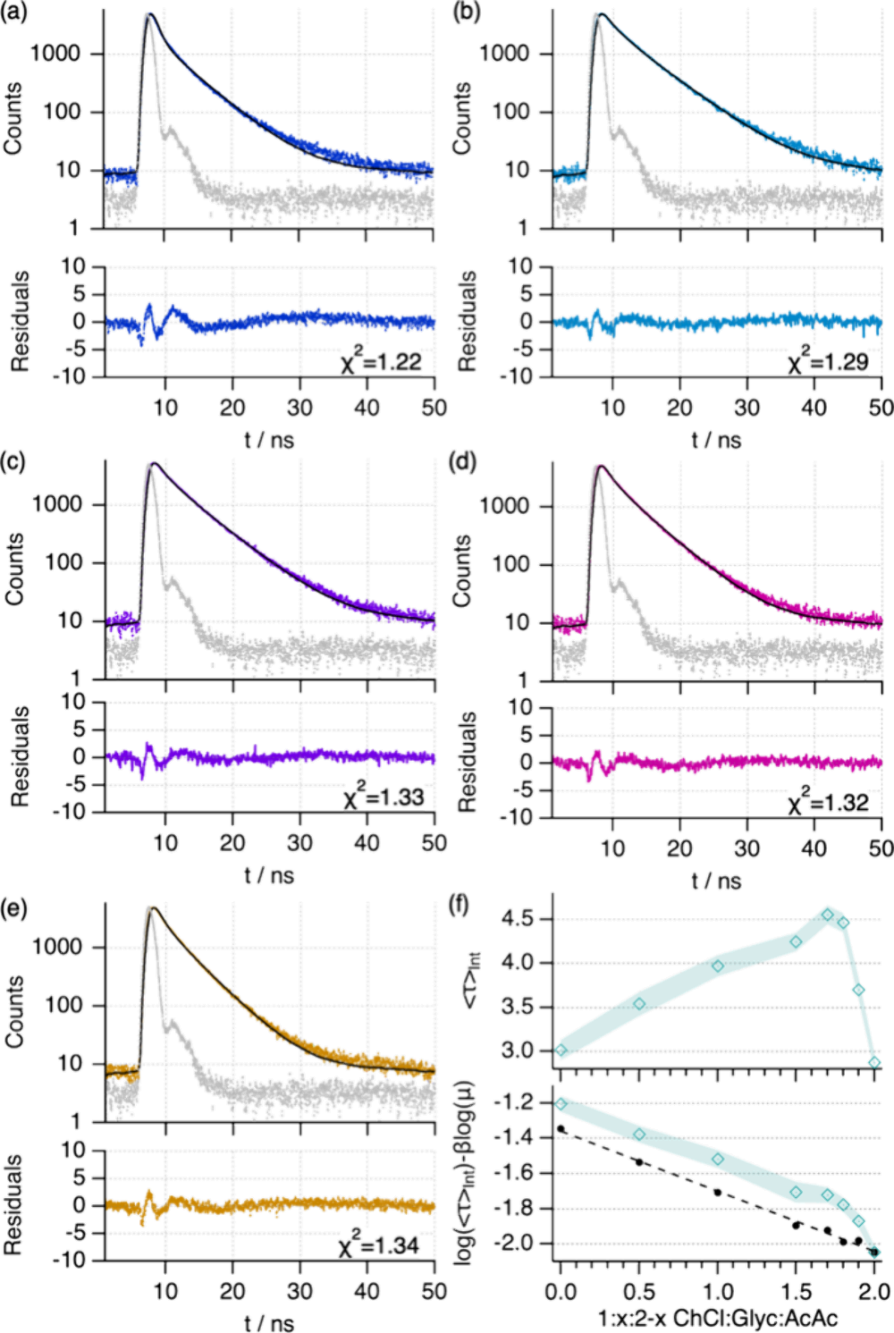
Excited-state emission fluorescence data, fits, and residuals of Lyz in 1:*x*:2–x ChCl:Glyc:AcOH at different HBD ratios in the DESs: (a) *x* = 2, (b) *x* = 1.5, (c) *x* = 1, (d) *x* = 0.5, and (e) *x* = 0. The instrument response function (IRF) is presented in each plot as gray dots. The residual panels present the χ^2^ values that quantify the fit’s quality. (f) The variation of the intensity-averaged fluorescence lifetime, <τ>_int_, and the Förster–Hoffmann representation of the viscosity-normalized lifetime values as a function of solvent composition. The dashed line in the Förster–Hoffmann representation shows the expected change in the lifetime when only the viscosity of the system changes.

The previous results confirmed that changes in the DES composition can be used to navigate the conformational landscape of the protein. However, we wondered if the protein resides in a discrete population of a specific folding intermediate or whether the transitions resulted from changes in the populations of different, coexisting conformational states. A suitable method for determining coexisting conformations is a transient emission technique, where the different energy relaxation pathways can be related to the fluorophores’ environment, for example, localization within the protein envelope or exposure to the solvent.^60^ As such, we performed time-correlated excited-state emission fluorescence (λ_ex_ = 280 nm) to investigate the possible coexistence of multiple folding states. The results show the characteristic decay associated with the relaxation of the Trp and Tyr residues of Lyz (λ_ex_ = 280 nm), where changes in their profile are observed with varying solvent composition. Initially, data were fitted using a double-exponential fluorescence decay model (Figure 7), as previously described for Lyz in an aqueous environment.^60^ Adding extra exponential decay did not significantly improve the quality of the fits (Figure S4 and Table S4). In parallel, we attempted to use a multiple-exponential model with fixed fluorescence lifetimes, where the data was fitted using weighted contributions from the folded and unfolded states as in a two-state unfolding mechanism (Figure S5 and Table S5).^61^ The results show that the use of a two-state model worsens the fits. As such, a far better agreement, parametrized by the quality of the fit χ^2^, was achieved when using two variable fluorescence lifetimes in a multistage unfolding mechanism. Therefore, these results confirm that the protein evolves through a landscape of discrete intermediate conformations with varying solvent compositions.

The extracted lifetimes also provided details in the unfolding pathway of Lyz, where the main differences appear in the slow component of the model (>3 ns), and only slight changes are observed in the fast lifetime (<1 ns). Using the intensity-averaged lifetime to compare the results, we observe a nonmonotonic variation of the fluorescence decay (Figure 7f). However, it must be considered that both the fluorophores’ environment and solvent viscosity change with varying solvent compositions (Table S6). To discern these effects, we determined the normalized lifetimes using a modified Förster–Hoffmann equation, which takes into account the influence of the viscosity in the fluorescence lifetime.^62,63^ This data representation reveals a multiple-stage transition with varying solvent compositions, agreeing with the previous characterization. For folded Lyz in 1:2 ChCl:Glyc, the normalized lifetime becomes slower than that in aqueous buffer (Figure S6).^60^ This change is potentially attributed to the higher viscosity of the DES and the concomitant slower rotamer interconversion of the aromatic residues.^36,64^ An increase in the normalized lifetime is observed when the AcOH component is present between 1.7 < *x* < 1.9, possibly attributed to small fluctuations in the internal structure of compactly folded Lyz according to our CD and SANS results (vide supra). At higher AcOH contents (0 < *x* < 1.5), the normalized lifetimes become faster due to the unfolding of the protein, where the gradual transition confirms the stabilization of different folding intermediates before finally reaching complete unfolding. Besides, the deviation from the expected trend when only viscosity effects are considered confirms the protein conformational changes. Therefore, our excited-state emission fluorescence results demonstrate that the protein resides in discrete folded states (instead of coexisting folded and unfolded states), which transition from compactly folded Lyz in glycerol-rich DESs to gradual unfolding with the increment in the AcOH content.

**Figure 8:**
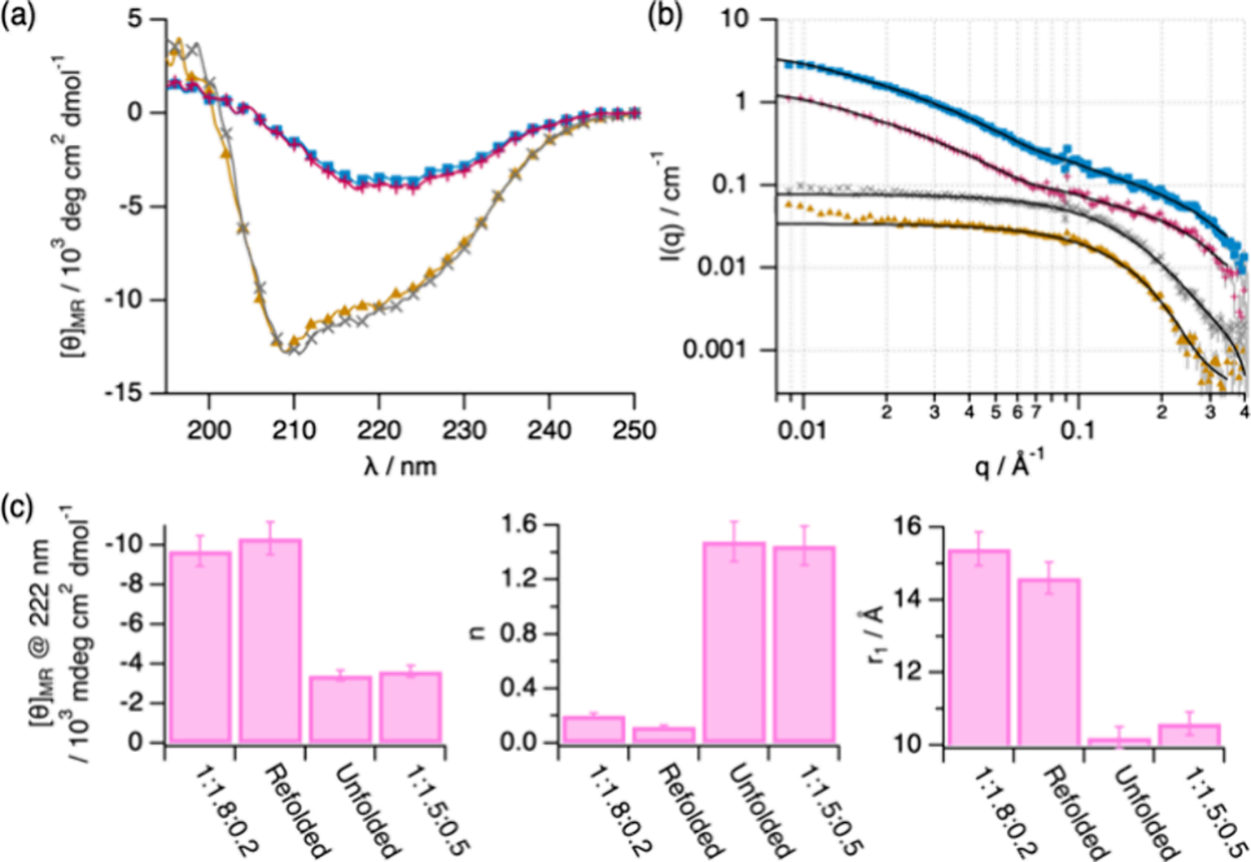
(a) Far-UV CD spectra and (b) SANS data and models for Lyz in 1:*x*:2–x ChCl:Glyc:AcOH ternary DESs, where the solvent composition was varied by adding the required amounts of 1:2 ChCl:Glyc or 1:2 ChCl:AcOH: 1:1.8:0.2 (brown triangle up solid), refolded (violet cross mark), unfolded (purple plus symbol), and 1:1.5:0.5 (blue square solid). Data and models in panel (b) have been offset for clarity by n*2. (c) The main results derived from the analysis of the spectroscopy and scattering data: mean residue ellipticity at 222 nm, Porod slopes at low *q* (n), and the apparent size of the protein monomer (*r*_1_). Where not seen, error bars are within the markers.

Once the stabilization of conformational intermediates was demonstrated, we sought to study whether those are locked states or the protein can still respond to variations in the solvent composition. To investigate this, Lyz was incorporated into a ternary DESs where it resides in an unfolded state (i.e., 1:1.5:0.5), and the composition of the solvent was varied by adding 1:2 ChCl:Glyc to reach a ratio where the protein is expected to regain a folded state (i.e., 1:1.8:0.2, labeled as refolded). The same protocol was followed to study the opposite transition, where the folded protein stabilized in a ternary DESs (i.e., 1:1.8:0.2) was mixed with 1:2 ChCl:AcOH to reach the unfolded state (i.e., 1:1.5:0.5, labeled as unfolded). This simple experiment reveals that the spectral and structural features of the protein can be driven in any direction by changing the composition of the solvent. When starting from the unfolded state, that is, 1:1.5:0.5 ChCl:Glyc:AcOH, the addition of 1:2 ChCl:Glyc to reach 1:1.8:0.2 ChCl:Glyc:AcOH prompts the protein to retrieve the same secondary structure and folding state (within error) to those observed when the protein is directly incorporated to the latter (Figure 8). Similarly, the addition of the acid–based binary DES to 1:1.8:0.2 ChCl:Glyc:AcOH to obtain 1:1.5:0.5 ChCl:Glyc:AcOH drives the protein from a (partially) folded state to an unfolded state. Also, it is observed that the low-*q* signal of the SANS data changes dramatically between the two states (Figure 8b). Thus, variations in the solvent composition can also modulate the collective behavior of the protein, confirming the formation or disassembly of transient clusters.

Our findings demonstrate that the compositional design of ternary DESs prompts gradual transitions in Lyz behavior that enable the capture of conformational intermediates, including folded, partially folded, and unfolded states. The overlap between the transitions in the secondary structure and the overall conformation suggests a cooperative folding mechanism, where the local topology of the protein backbone possibly controls the folding state of Lyz. Concomitantly, the loss of a globular conformation leads to an increased degree of interchain interactions, which are mediated by the folding states of the protein. No irreversible changes are caused to the protein; either unfolding or refolding can be attained through variations in the solvent composition, allowing for the dynamic exploration of the conformational landscape. This is highly relevant for the unfolded states in ternary DESs, where refolding and disassembly of transient clusters can still be prompted. In contrast, proteins in aqueous environments tend to self-associate in nonspecific hierarchies in the absence of chaperones, often leading to irreversible aggregation.^65^ Thus, these experiments reveal not only that the structure of Lyz can be controlled by changing the composition of DES but also that it can be used to modulate the collective behavior of the protein.

### Formation of Protein Eutectogels

**Figure 9:**
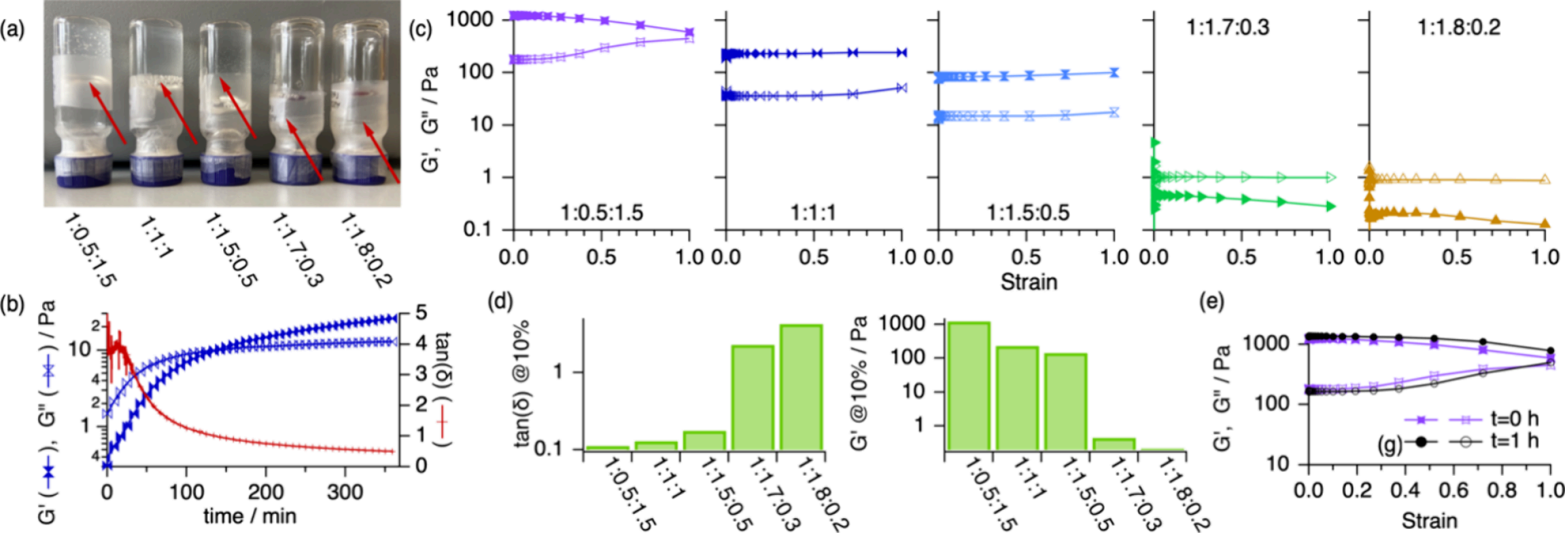
Rheological investigation of the samples containing Lyz in 1:0.5:1.5, 1:1:1, 1:1.5:0.5, 1:1.7:0.3, and 1:1.8:0.2 ChCl:Glyc:AcOH. (a) Vial inversion test, where the red arrows indicate the position of the sample inside the vial. (b) Gelation curve depicting the evolution of the storage modulus (*G*′, filled markers) and loss modulus (*G*″, open markers) as a function of time for the sample containing 42 mg/mL Lyz in 1:1:1 ChCl:Glyc:AcOH immediately after preparation. Sampling was performed every 10 s at a frequency of 1 Hz and 0.1 strain. (c) Strain sweeps showing the *G***′** (filled markers) and *G*″ (open markers) of the Lyz solutions in compositionally varied DESs, as shown in the legend of the panels. From the moduli, the characteristic rheological parameters were determined: (d) the loss factor (tan(δ)) and elastic modulus at 10% strain. (e) The healing test of two strain sweep cycles separated by 1 h of the gel in 1:0.5:1.5 ChCl:Glyc:AcOH.

Previous investigations have shown the formation of eutectogels based on polymers,^66^ small molecules,^67^ peptides,^68,69^ and fibrous proteins.^41^ However, the formation of eutectogels based on proteins with controlled topology remained elusive until now. Having established a direct connection between protein behavior and the solvent’s characteristics, we then explore the rational design of protein eutectogels. As the degree of unfolding in the presence of chemical denaturants correlates to the mechanical response upon gel formation,^8,40,70^ the realization of protein-based eutectogels with controlled properties constitutes an obvious proxy for applying these triggered folds into biomaterials. Thus, we explored the low-temperature formation of DES-based gels (eutectogels) using Lyz to prove this. Samples were prepared at a Lyz concentration of 42 mg/mL, and the composition of the ternary DESs was systematically varied to attain different degrees of protein folding, that is, globular, partially folded, and unfolded. All samples showed a transparent appearance after incubation, and the vial inversion test was used to assess gel formation (Figure 9a). At the ratios 1:1.5:0.5, 1:1:1, and 1:0.5:1.5 ChCl:Glyc:AcOH, sample gelation was observed, whereas at higher glycerol contents (1:1.65:0.35 and 1:1.8:0.2), the sample did not form a gel. The gelation process was confirmed by following the evolution of the storage (*G*′) and loss (*G*″) moduli, which respectively describe the elastic and viscous components of the system,^70^ immediately after the preparation of a sample in 1:1:1 ChCl:Glyc:AcOH (Figure 9b). The results show a gradual progression of the two moduli, characterized by a sharp increase at short times (<100 min) followed by a gradual increase of *G*′ at longer times. Importantly, *G*′ becomes dominant over *G*″ after 130 min, causing a reversion in the loss ratio (tan(δ)). At this stage, gel formation begins.

The rheological properties of the system as a function of solvent composition were investigated using strain-controlled frequency sweeps experiments to determine *G*′ and *G*″. Samples in 1:1.5:0.5, 1:1:1, and 1:0.5:1.5 ChCl:Glyc:AcOH show a dominant *G*′ with a loss factor around 0.1, confirming the formation of gels (Figure 9d). The absence of a crossover point over the entire strain range on the strain-sweep experiments suggests the formation of stiff gels with a resilient elastic response (Figure 9c). In contrast, the samples in 1:1.7:0.3 and 1:1.8:0.2 ChCl:Glyc:AcOH behave as viscous fluids instead of gels for which *G*″ is higher than *G*′ over the entire strain range. The *G*′ at 10% strain was used to compare the mechanical properties of the gels, showing that these vary with solvent composition. The elastic component of the gel in 1:0.5:1.5 ChCl:Glyc:AcOH is 6- and 9-fold higher than that in 1:1:1 and 1:1.5:0.5 ChCl:Glyc:AcOH, respectively, and about 3000-fold higher than that from nongelled samples. It should be noted that this particular rheological response does not arise from the viscosity of the DESs; in fact, the viscosity of 1:2 ChCl:Glyc is about 10-fold higher than that of 1:2 ChCl:AcOH (Table S6). In addition, we investigated the self-healing properties of the gel. This study was performed for Lyz in 1:0.5:1.5 ChCl:Glyc:AcOH as some yield can be observed above 50% strain, thus causing significant physical deformation to the gel (Figure 9e). After running a frequency sweep up to 100% strain, the system was left to relax, and the rheology of the system was measured after 1 h (Figure 9f). The results show that the values of *G*′ and *G*″ from the two stress cycles almost overlap across the entire strain range, with only a subtle hardening in the second cycle. This overlap confirms the recovery of the gel structure upon rest.

Our results reveal a key idea: the composition of the ternary DESs can be tailored to control protein folding, and the resulting conformational state yields different macroscopic responses. The degree of protein unfolding attained at high acid-based DES contents results in the formation of mechanically stronger gels. In contrast, intermediate folds with weaker collective interactions result in lower moduli. The gradual evolution of the rheological properties with time suggests that gelation can be attributed to the entanglement of unfolded protein chains into a three-dimensional network defined by the topological interactions (excluded volume) between the chains.^71^ As expected, the system does not show gel properties despite the high protein concentration when the protein resides in a globular conformation, that is, low acid-based DES ratios.

## Conclusions

The long-standing paradigm of protein behavior in aqueous solution is that the structure and dynamics of the protein dictate its stability. When the so-called native state is perturbed, protein stability is threatened.^65,72,73^ Inspired by this frontier, we investigated how anhydrous DESs can be designed to trap conformational intermediates of proteins selectively. Our results demonstrate that varying the solvent composition can stabilize a given protein’s globular, partially folded, and unfolded conformation. Lyz was found to be more structurally resilient, with subtle differences in the secondary, tertiary, and quaternary structure compared to the native state in 1:2 ChCl:Glyc, 1:2 ChAcO:Glyc, and 1:2 ChCl:Urea. Only 1:2 ChCl:AcOH resulted in Lyz unfolding. In contrast, the native conformation of BSA was altered in all of the DESs. The glycerol-based DESs resulted in partially folded conformations with increased self-association, whereas the urea- and acid-based DESs led to immediate denaturation and aggregation. Therefore, the protein behavior in anhydrous DESs depends on the characteristics of both the protein and the solvent, where changes in the hydrogen bond donor are particularly important at triggering protein denaturation.

The design of ternary DESs, obtained by mixing two DESs sharing the same organic salt and different hydrogen bond donors, allows us to fine-tune the Lyz conformation. In such a case, DES composition dictates the folding state of the monomer, varying between a native-like conformation and fully unfolded chains. Also, the collective behavior of the protein can be tuned through these changes, yielding clustering or entanglement on demand. These transitions are entirely reversible and without threatening colloidal stability, where the protein undergoes refolding and unfolding processes by varying the DES constituents. These intermediate folds and unfolded states of Lyz can be prompted to entangle and form gels, forming conformationally triggered protein-based eutectogels. Notably, the mechanical properties of the gels are defined by the conformation of the protein, which in turn depends on the solvent composition. Gelation can be easily achieved without requiring covalent cross-linking or other chemical denaturants, and the resulting gel showed self-healing properties upon deformation.

In recent years, DESs have emerged as alternative solvents for protein preservation in nonaqueous environments, drug delivery vectors, and the development of biomaterials.^67,68,74,75^ As the concept of “designer” solvents encompasses the selection of particular physicochemical properties through changes in the solvent, understanding protein behavior in DESs becomes essential to realize these as functional environments. We have shown how DESs can modulate protein behavior, resulting in biomaterials with a close connection between the protein conformation and function. Indeed, a more sophisticated molecular model is required to understand the interactions that stabilize the different protein folded states fully. However, further developments in MD simulations of non-native folds in DESs are required, as we are still in the infancy of molecular simulations of proteins in these complex solvents.^37,76^ Overall, there is enormous scope for further research in adapting proteins to fulfill specific functions through tailoring solvent properties.

## Methods

### Deep Eutectic Solvent Preparation

All precursors for DES preparation were dried in a vacuum oven before solvent preparation (except for acetic acid). Protiated and deuterated DESs were prepared under an argon atmosphere by mixing the required precursors at the eutectic ratio (i.e., 1:2 molar ratio), followed by stirring and mild heating at 50 or 60 °C until a clear liquid was formed. The ternary solvents were prepared by mixing amounts of 1:2 ChCl:Glyc and 1:2 ChCl:AcOH to yield the required ratios, followed by equilibration at room temperature. Once ready, the resulting solvents were stored sealed and under a dry atmosphere to avoid water adsorption.

### Protein Incorporation to Anhydrous DESs and Aqueous Buffer

Samples containing protein were prepared by mixing a concentrated aqueous stock solution of the proteins with the required amount of DES. The samples were subsequently freeze-dried on an Epsilon 2–6D LSCplus instrument from Martin Christ with controlled temperature and pressure to avoid protein degradation. The residual water in the samples was measured using Karl Fischer coulometric titration to an average water content of 0.24% for 1:2 ChCl:Urea, 0.47% for 1:2 ChCl:Glyc, 0.42% for 1:2 ChAcO:Glyc, and 0.63% for 1:2 ChCl:AcOH. Samples in aqueous buffer were prepared using 10 mM, pH 7 phosphate buffer, and Mili-Q water. For the samples in D_2_O, the 10 mM phosphate buffer was adjusted to pH 7.4 according to Rubinson’s protocol.^77^ The final protein concentration was determined in each sample using an ND-1000 Spectrophotometer (Saveen Werner).

### UV–Vis Spectroscopy

Measurements were performed on a Varian Cary 50 UV–Vis spectrometer. Samples were loaded in a 1 mm quartz Hellma cell and placed on the sample stage at 25 °C. Protein concentration was 100 μM. The solvent absorption contribution was subtracted from each sample’s signal. Second-derivative UV spectra were determined by applying a first derivative to the data, passing a Gaussian filter to the new spectra, and performing the second derivative of the smoothed spectra.

### Fluorescence Spectroscopy

Steady-state fluorescence spectroscopy measurements were performed on a Cary Eclipse fluorescence Spectrometer using a 96-well plate sample stage. The excitation wavelength was 295 nm, and emitted intensity was collected between 300 and 600 nm with the excitation and emission slits at 5 nm. The scan rate was 600 nm/min, and the data were averaged for 15 scans. Protein concentration was 10 μM. After subtraction of the solvent signal, the data were normalized to the maximum emitted intensity.

Excited-state fluorescence emission intensity experiments were conducted on an FS5 Spectrofluorometer from Edinburgh Instruments equipped with a fixed pulse LED source. The excitation wavelength was 284 nm, with a bandwidth of 9.9 nm and a pulse width of 794.7 ps. Samples were loaded in 10 mm path length, 4 mm width quartz cuvettes. Data were collected over 50 ns after each pulse, with a pulse repetition of 100 ns and an increment rate of 0.024 ns/channel. Lysozyme concentration was 20 μM.

### Circular Dichroism

CD measurements were performed on a Jasco J-715 instrument with a temperature-controlled sample stage. Samples were loaded in a 0.1 mm quartz Hellma cell and placed on the sample stage at 25 °C. Data were acquired at a scan rate of 50 nm/min, with a spectral bandwidth of 1 nm and 1 s response time, and accumulated for three scans. Protein concentrations were 240 and 60 μM for lysozyme and BSA, respectively. The contribution from the solvents was subtracted from each sample. The mean residue ellipticities were calculated from the ellipticity measured and the protein concentration.

The spectrometers were equipped with a temperature-controlled sample stage, and measurements were performed at 25 °C.

### Density and Viscosity Measurements

The densities of the DESs (no protein added) were determined using the vibrating U-tube method on a DMA-5000 by Anton Paar at 25 °C The viscosities of the binary and ternary DESs were determined by the micro Ubbelohde viscometer technique at 25 °C using the calibrated glass capillaries IIc and III. The automated flow measurements were performed using a Lauda Processor Viscosity System PVS1 with a resolution of 0.01 s.

### Small-Angle Neutron Scattering Measurements and Analysis

Samples for SANS experiments were prepared using deuterated solvents, that is, 1:2 choline-*d*_9_ chloride:glycerol-*d*_8_, choline-*d*_9_ acetate-*d*_3_:glycerol-*d*_8_, 1:2 choline-*d*_9_ chloride:urea-*d*_4_, 1:2 choline-d_9_ chloride:acetic acid-d_4_, buffered D_2_O, and protiated proteins. Protein concentration was 240 and 60 μM for lysozyme and BSA, respectively. SANS experiments were performed on D22 and D33 at Institut Laue-Langevin under experiment numbers 8-03-1049 and 9-13-1062, respectively.^78,79^ Samples were loaded in 1 mm path length, 1 cm width cuvettes, and the temperature was kept constant at 25 °C. Data were reduced using Grasp^80^ and analyzed using the indirect Fourier transform method implemented in the ATSAS 3.0 suite,^46^ and power-law analysis was performed using SasView 5.0.

### Gelation Experiments

Lysozyme samples in selected ternary DESs were prepared at a final protein concentration of 42 mg/mL (0.6 mM) final protein concentration. A lysozyme stock solution in 1:2 ChCl:Glyc at 140 mg/mL (2.1 mM) was mixed with 1:2 ChCl:Glyc and 1:2 ChCl:AcOH to obtain the required molar ratios in the solvents. Samples were heated to 40 °C for 10 min to facilitate homogenization and allowed to equilibrate at 25 °C for 12 h. A vial inversion test was initially employed to assess the gel formation.

The kinetics of the gel formation were acquired at 25 °C for a freshly prepared sample of 42 mg/mL Lyz in 1:1:1 ChCl:Glyc:AcOH. Immediately upon mixing, *G*′ and *G*″ were monitored for 360 min at a constant frequency of 1 Hz and constant strain of 10% with six measurements per minute. The mechanical characterization of the samples was performed on an Anton Paar MCR 301 stress-controlled rheometer using a 25 mm cone–plate configuration with a 1° angle and a 0.048 mm gap. Strain sweeps were performed at a constant frequency of 1 Hz over the strain range of 0.01–100% with seven measurements per decade.

To prevent water adsorption, silicon oil was placed around the edge of the geometry, which should not contribute to the measurement’s systematic error due to the oil’s low viscosity. Experiments were conducted at 25 °C.

## Data Availability

Data are openly available at 10.5281/zenodo.10521608.

## Supporting Information Available

Details on materials, characterization techniques, and data analysis; results from the characterization of proteins in binary DESs (Table S2 and S3); validation of the exponential component analysis from excited-state emission fluorescence (Figure S4 and Table S4); analysis of the excited-state emission fluorescence data using the two-state denaturation model (Figure S5 and Table S5); exited-state emission fluorescence results of lysozyme in aqueous buffer (Figure S6); densities and viscosities of the DESs (Table S6); supporting references^81−85^ (PDF)

## Notes

Data are openly available at 10.5281/zenodo.10521608.
