# Supplementary material for: Stabilization of Non-Native Folds and Programmable Protein Gelation in Compositionally Designed Deep Eutectic Solvents
Source: ACS Nano. 2024 Jul 1;18(28):18314–26. doi: 10.1021/acsnano.4c01950 (PMC11256765; doi:10.1021/acsnano.4c01950)
Supplement: Supplementary file 1 — nn4c01950_si_001.pdf [file nn4c01950_si_001.pdf]

Supplementary Information for:
Stabilization of non-native folds and programmable protein gelation in compositionally designed deep eutectic solvents

Adrian Sanchez-Fernandez,[a]* Jia-Fei Poon,[b] Anna E. Leung,[b] Sylvain F. Prévost,[c] Cedric Dicko.[d,e]
[a]Centro Singular de Investigación en Química Biolóxica e Materiais Moleculares (CIQUS), Universidade de Santiago de Compostela, Rúa de Jenaro de la Fuente, s/n, 15705 Santiago de Compostela, Spain.
[b]European Spallation Source, Lund University, Box 176, SE-221 00 Lund, Sweden.
[c]Institut Laue-Langevin, DS / LSS, 71 avenue des Martyrs, 38000, Grenoble, France.
[d]Pure and Applied Biochemistry, Department of Chemistry, Lund University, Box 124, SE-221 00 Lund, Sweden.
[e]Lund Institute of Advanced Neutron and X-ray Science, SE-223 70 Lund, Sweden.
*adriansanchez.fernandez@usc.es

Table of contents

1 Materials and methods 2
1.1 Materials 2
1.2 Synthesis and characterization of choline-d_9 acetate-d_3 2
1.3 Sample preparation 4
1.4 Methods 4
1.5 Data analysis 5
2 Results from the characterization of the proteins 7
3 Validation of the exponential component analysis from excited-state emission fluorescence 7
4 Analysis from the two-state denaturation model 8
5 Excited-state emission fluorescence of lysozyme in aqueous buffer 8
6 Densities and viscosities of the DESs 9
7 References 9

1 Materials and methods

1.1 Materials

Bovine serum albumin (BSA, fatty acid free, >98%) and chicken egg white lysozyme (Lyz, >98%) were purchased from Sigma and used as received. Choline chloride (ChCl, Sigma, >99%), choline acetate (ChAc, Iolitec, >98%), urea (Urea, Sigma, 99.0-100.5%), glycerol (Glyc, Sigma, >99.5%%), anhydrous acetic acid (AcOH, Sigma, >99%), sodium phosphate monobasic dihydrate (Sigma, >99.5%%), sodium hydroxide (Sigma, >99%), and D_2O (Sigma, 99.9 %D) were used without further purification. Choline-d_9 chloride (d-ChCl, 98 %D), urea-d_4 (d-Urea, 98 %D), glycerol-d_8 (d-Glyc, 99 %D), acetic acid-d_4 (d-AcOH, 99.5 %D), and sodium acetate-d_3 (99 %D) were supplied by Cambridge Isotope Labs and used without further purification. Choline-d_9 acetate-d_3 (d-ChAc) was synthesized using the protocol explained below. Water used for sample preparation was of Milli-Q grade, resistivity 18.2 MΩ cm at 25 °C.

The chemicals used for DES preparation were vacuum dried at 70 °C (ChCl, d-ChCl, Urea, and d-Urea) or 50 °C (ChAc, d-ChAc, Glyc, and d-Glyc) for 24 hours before preparation of the DES. AcOH and d-AcOH were not vacuum dried due to the high vapor pressure of the compound. The different DESs (1:2 ChCl:Urea, 1:2 ChCl:Glyc, 1:2 ChAc:Glyc, 1:2 ChCl:AcOH, and their deuterated analogues) were prepared by weighing the required amounts of each chemical into a round bottom flask, followed by continuous stirring and heating at 60 °C under an argon atmosphere until a clear liquid was formed. After cooling, the DESs were equilibrated at room temperature in a desiccator for a minimum of 24 h before use. Once ready, the DESs were stored sealed and under a dry atmosphere to avoid water adsorption. The residual water in the DESs was measured using Karl-Fischer titration to an average water content of 0.24% for 1:2 ChCl:U, 0.47% for 1:2 ChCl:G, 0.42% for 1:2 ChAc:G, 0.63% for 1:2 ChCl:AcOH.

Phosphate buffer (10 mM, pH 7 in water and 10 mM, pH 7.4 in D_2O) was used as reference for the protein state in aqueous buffer (from herein referred to as native state) in the experiments presented here.

1.2 Synthesis and characterization of choline-d_9 acetate-d_3

Choline chloride (trimethyl-d_9) (98% isotopic purity) and sodium acetate-d_3 (99% isotopic purity) were used without further purification. All other reagents and solvents were purchased from Sigma-Aldrich. NMR spectra were recorded on a Varian Unity INOVA 400 MHz spectrometer or a Bruker Avance Neo 500 MHz spectrometer. ^{13}C NMR spectra were ^{1}H-decoupled. Spectra were recorded at 298 K. Chemical shifts, expressed in parts per million (ppm), were referenced to the residual signal of the solvent.

Choline (trimethyl-d_9) acetate-d_3

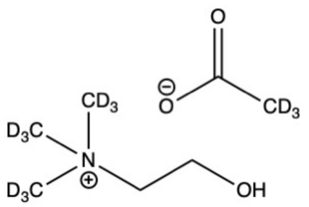

Using a modification of a reported procedure,[1] a solution of sodium acetate-d_3 (1.43 g, 16.8 mmol) in methanol/absolute ethanol (67.5 mL:35 mL) was added to a solution of choline chloride (trimethyl-d_9) (2.50 g, 16.8 mmol) in methanol (67.5 mL) and the mixture was stirred for one hour at room temperature. Sodium chloride precipitated and was removed by filtration. Acetone (50 mL) was added after which a second crop of sodium chloride precipitated and was removed by filtration. The solvents were removed *in vacuo* to provide the title compound as a clear, viscous liquid (2.35 g, 80%). ^{1}H NMR (500 MHz, CD_3OD) δ 3.47 (m, 2H), 4.00 (m, 2H). ^{13}C NMR (125 MHz, CD_3OD) δ 53.7 (m), 57.0, 68.8, 180.2. ^{1}H NMR (400 MHz, D_2O) δ 3.47 (m, 2H), 4.01 (m, 2H). This is in accordance with the relevant reported data for the protiated isotopologue.[1]

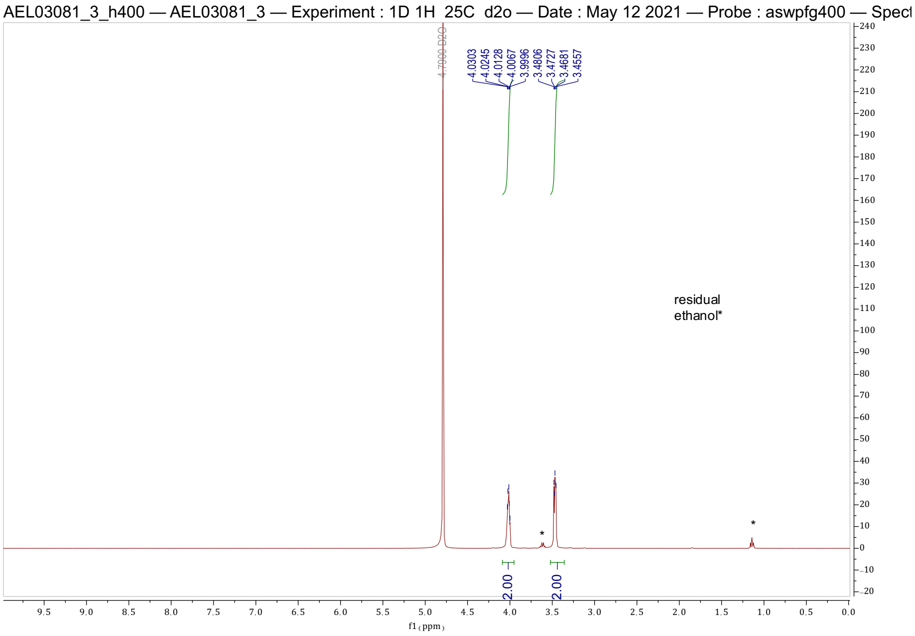

Figure S1 ^{1}H NMR spectrum of choline (trimethyl-d_9) acetate-d_3 (400 MHz, D_2O).

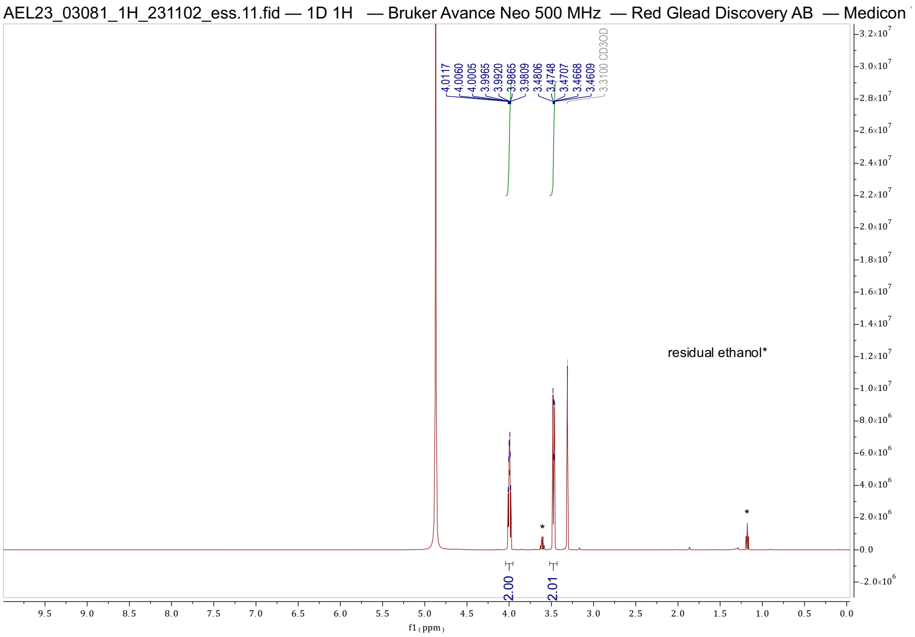

Figure S2 ^{1}H NMR spectrum of choline (trimethyl-d_9) acetate-d_3 (500 MHz, CD_3OD).

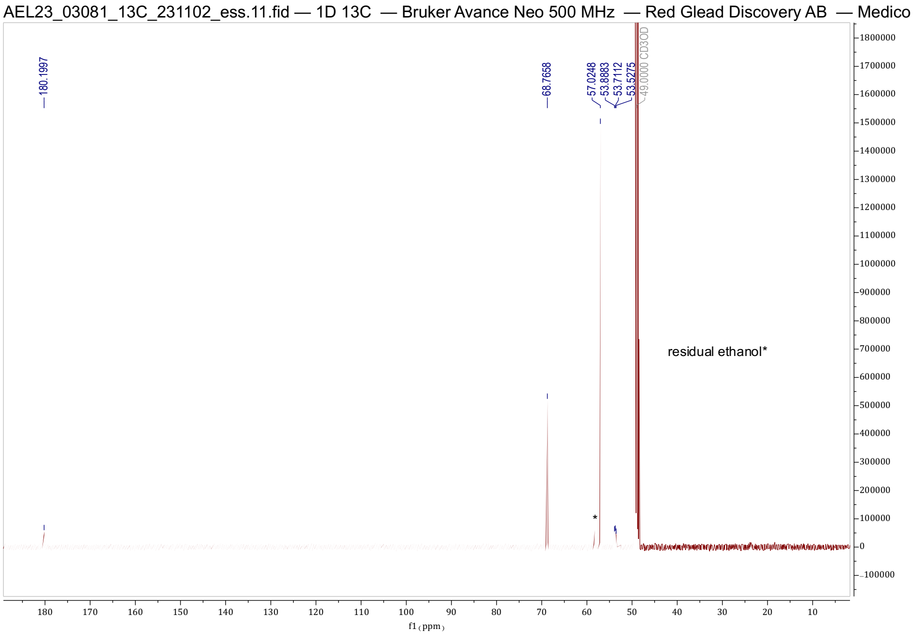

Figure S3 ^{13}C NMR spectrum of choline (trimethyl-*d*$_9$) acetate-*d*$_3$ (125 MHz, CD_3OD).

1.3 Sample preparation

Initially, protein stock solutions were prepared in water or D_2O by adding the required amounts of BSA or Lyz to water for a protein concentration of *ca.* 5000 µM. 10- and 50-fold dilutions of the 1000 µM samples were conducted to obtain the stock solutions at lower concentrations, 500 and 100 µM respectively. Each protein solution was then centrifuged at 13 000 rpm for 5 min at 25 °C to remove any aggregates from the solution, followed by pipetting of the supernatant into new tubes.

Protein samples in neat (anhydrous) DESs for spectroscopic characterization were prepared by mixing the required amount of each DES and protein stock solution (non-buffered) in a glass vial. The added amount of protein stock solution was at least 20 times lower (v/v) than the added amount of DES to minimize the initial water content. Subsequently, samples were freeze-dried using an optimized protocol as explained below. Aqueous protein solutions were prepared in phosphate buffer following the same dilution protocol. Samples were stored at 4 °C between preparation and measurements and used within 72 hours after preparation to minimize the impact of protein degradation on the results.

The samples for the SANS experiments were prepared in deuterated DESs and buffered D_2O using the protocol presented for the protiated analogues. These samples were stored at -20 °C before measurement and used within a week of sample preparation.

The samples at high protein concentration required for the gelation experiments were prepared from highly concentrated, aqueous solutions of Lyz, i.e., 350 mg mL^{-1}. The aqueous stock solution was then centrifuged at 4 500 rpm for 5 min at room temperature to remove any aggregates. Then, 1:2 ChCl:Glyc was added to a final concentration in the stock DES (after water removal) of 140 mg mL^{-1} and water was subsequently lyophilized to yield the stock solution in DES. Samples were prepared by mixing the stock solution with 1:2 ChCl:Glyc and 1:2 ChCl:AcOH so that the required ratios in the system 1:x:2-x ChCl:Glyc:AcOH were reached at a final protein concentration that was a 3-fold dilution of the stock solution in DES.

Control samples of the DESs without protein submitted to the same protocols were tested throughout the research activates for water content using Karl-Fischer titration. The results from the titration showed minor variations in the water content (±0.2 wt%) during this process. The water average water content was 0.24% for 1:2 ChCl:Urea, 0.47% for 1:2 ChCl:Glyc, 0.42% for 1:2 ChAc:Glyc, and 0.63% for 1:2 ChCl:AcOH.

1.4 Methods

Freeze-drying was performed on an Epsilon 2-6D LSCplus from Martin Christ to remove the water previously added during protein incorporation to the DESs. The samples were loaded in glass vials and introduced in the instrument tray. Initially, the samples were quenched to -40 °C by abruptly lowering the temperature of the trays. Once the samples reached the target temperature and solidify, the pressure in the instrument was gradually reduced to 0.8 mbar to sublimate the water in the sample while the temperature was gradually risen to -15 °C for 24 hours. Subsequently, the temperature of the system was adjusted to room temperature. Finally, the instrument was automatically closed the vials under dry atmosphere and the system was vented to reach ambient conditions. Using this protocol avoids the sample bubbling which could lead to protein degradation during freeze-drying stages.

The final protein concentration in the samples was determined using a ND-1000 Spectrophotometer from Saveen Werner by measuring the absorbance at 280 nm using the protein extinction coefficient (BSA: ε=43,824 cm^{-1} M^{-1}, M=66,400 g mol^{-1}; Lys: ε=38,940 cm^{-1} M^{-1}, M=14,300 g mol^{-1}). Corrections for solvent contribution were performed for each sample.

UV-Vis absorption measurements were performed using the Varian Cary 50 UV-Vis Spectrometer. Samples were loaded in 1 mm light path, 10 mm width Quartz. The absorbance was measured in the range 190-500 nm at 600 nm min^{-1}. The contributions from the solvents were subtracted to each sample. The first-derivative and second-derivative UV spectra were determined in a wavelength range between 240 and 310 nm. The peaks in the tyrosine/tryptophan region (280-300 nm) in the second-derivative UV spectra were analyzed to extract the positions and amplitudes of the peaks as previously described.[2]

Steady-state fluorescence measurements were carried out on a Cary Eclipse Fluorescence Spectrometer in the 96-well plate configuration at excitation wavelengths of 295 nm (Trp). The emitted intensity was measured between 300-600 nm at 600 nm min^{-1} using excitation and emissions slit of 5 nm. Data were accumulated for 15 scans for each measurement.

Exited-state emission fluorescence measurements were conducted on a FS5 Spectrofluorometer from Edinburgh Instruments. The source was a fixed pulsed LED (pulse width 794.7 ps) with an excitation wavelength of 280 nm, thus targeting the Tyr and Trp residues of the protein. Data were acquired using 1-cm path length, 4-cm width, quartz cuvettes with the samples loaded on a temperature-controlled sample stage at 25 °C. The emitted intensity was collected for 50 ns after each pulse with a resolution of 0.0244 ns. The evolution of the emitted intensity with time was collected at 345 nm and data were accumulated to reach 5000 counts at the main pulse to obtain an appropriate signal-to-noise ratio. The instrument response function (IRF) was obtained using the scattering solution of 0.1 wt% SiO_2 (LUDOX® HS-30) particles in water.

Far-UV circular dichroism (CD) spectroscopy was acquired using a was performed on a Jasco J-715 between 190 nm and 250 nm at 50 nm min^{-1}. A spectral bandwidth of 1 nm and a response time of 1 s were used, and data were accumulated for 10 scans. Samples were loaded in a 0.1-mm quartz cuvette. The UV-vis absorption of each sample at 280 nm were used to determine the protein concentration and effective pathlength. The contribution from each was subtracted to each spectrum. The mean residue ellipticity ($[\theta]_{MR}$) was calculated as follows:

$$[\theta]_{MR} = \frac{\theta}{nl[protein]}$$

where θ is the measured ellipticity in deg, n is the number of amino acid residues (583 for BSA, 113 for Lyz), l is the cuvette path length in cm^{-1}, and [protein] is the concentration of either BSA or Lyz in the sample in dmol cm^{-3}.

All spectrometers were equipped with a temperature-controlled sample stage and measurements were performed at 25 °C.

Small-angle neutron scattering (SANS) measurements were performed on D22 and D33 at the Institut Laue-Langevin (Grenoble, France). Sample were loaded in 1 mm path length, 1 cm width, quartz Hellma cells and placed in a temperature-controlled sample changer at 25 °C for measurement. On D22, the sample-to-detector distances were 1.7 m and 8 m for the front and rear detectors, respectively. Measurements were conducted using a neutron wavelength of 6 Å. This setup provided a combined q-range of 0.006-0.65 Å^{-1}. The detectors of D33 were placed at 1.7 m and 8 m from the sample and data were collected using a neutron wavelength of 6 Å. This configuration yielded a combined q-range of 0.0047-0.34 Å^{-1}. The raw data was reduced according to the instruments' protocols using the GRASP V10.17i software,[3] using the attenuated direct beam to obtain intensities in absolute scale. The contribution from the empty cell was subtracted, and the noise was accounted for by subtracting the signal recorded with a sintered B4C absorber. In addition, the scattering from the solvent was subtracted accounting for the incoherent contribution observed at high q values. The reduced data is presented as scattered intensity in absolute scale, I(q) in cm^{-1}, versus momentum transfer, q in Å^{-1}.

Densities (ρ) of the DESs were measured using a vibrating U-tube densimeter DMA-5000 by Anton Paar at 25 °C. The uncertainty in density is estimated to be 0.001 g cm^{-3}. The kinematic viscosity (ν) of the binary and ternary DESs was determined using a capillary-based method in a micro Ubbelohde viscometer. Samples were loaded in the Schott capillaries IIc and III, and the automated Lauda Processor Viscosity PVS1 system determined the flow time with a resolution of 0.01 s. The temperature of the system was kept constant at 25 °C. Three repeats were performed per sample and the error bar corresponds to the standard deviation between these measurements. From the kinematic viscosity and the density values, the dynamic viscosity (η) is given by the formula:

$$\eta = v\rho = Kt\rho$$

The rheology measurements were carried out on an Anton Paar MCR 301 at 25 °C using a cone plate with a diameter of 25 mm, a cone angle of 1° and a gap of 0.048 mm. Strain sweeps of samples containing Lyz (42 mg mL^{-1}) in DES were performed at constant frequency of 1 Hz over the strain range of 0.01 –100% with 7 measurements per decade. The kinetics of the gel formation at 25 °C were determined by monitoring the evolution of the storage moduli G'. A sample was prepared freshly by mixing 350 µL of Lyz solution (84 mg mL^{-1}) in 1:2 ChCl:Glyc with 350 µL of 1:2 ChCl:AcOH. The sample was gently mixed for a few seconds and placed on the rheometer. G' was collected for 360 min at constant frequency of 1 Hz and strain of 10% with 6 measurements per minute. A small amount of low viscosity silicon oil was placed around the geometry to avoid water absorption during measurements.

1.5 Data analysis

The analysis of the time-correlated single photon counting obtained from the exited-state emission fluorescence was performed in the FAST software package from Edinburgh Instruments. The analyses were conducted using a reconvolution

approach, by which the theoretical models were iteratively convoluted with the instrument response function. The theoretical models were determined using the exponential components analysis, as described in the following equation:

$$I(t) = B + \sum_{i=1}^{n} \alpha_i e^{\frac{t}{\tau_i}}$$

where B is the background signal, τ_i is the decay time, and α_i is the weighing of each decay component i. Initially, models containing one, two, or three exponential decays were tested. However, the best results were obtained from the double-exponential decay function, as the addition of an extra component did not significantly improve the quality of the fit. This observation is in good agreement with previous reports.[4] Thus, we concluded that the systems only can be described using two characteristic decay times and the equation was reduced to:

$$I(t) = B + \alpha_1 e^{\frac{t}{\tau_1}} + \alpha_2 e^{\frac{t}{\tau_2}}$$

The quality of the fits was evaluated by the calculation of the χ^2 statistical parameter using the formula:

$$\chi^2 = \sum_{k=1}^{m} \frac{[I(t_k) - I_c(t_k)]^2}{I(t_k)}$$

where $I(t_k)$ and $I_c(t_k)$ represent the measured and calculated emitted intensities at time *k* respectively.

From the calculated populations of each decay, the intensity average lifetime, $<\tau>_{int}$, was determined as the main changes were observed in the slow component of the decays. This approach weighs the lifetime of each component by the intensity of that component using the equation:

$$< \tau >_{int} = \frac{\sum_{i=1}^{n} \alpha_i {\tau_i}^2}{\sum_{i=1}^{n} \alpha_i \tau_i}$$

The average lifetimes were normalized to the viscosity of the continuum using the modified Förster–Hoffmann equation:[5]

$$< \tau >_{int} = \frac{z\eta^{\beta}}{k_r}$$

Or in the logarithmic form:

$$log(< \tau >_{int}) = log\left(\frac{z}{k_r}\right) + \beta log(\eta)$$

where *z*, k_r, and β represent constants related to the decay mechanism. For a fluorophore with no changes in the decay mode, e.g., fluorophore in water-glycerol mixtures,[5] the $log(<\tau>_{int})$ and $log(\eta)$ are expected to follow a linear relationship. However, changes in the environment of the fluorophore, e.g., conformational change of the protein, could lead to deviations from linearity.

SANS data were analyzed using a combination of model-free methods that yield information on the shape and size of the protein. The pair-distance distribution function of the scatterer (*p(r)*) were determined using the indirect Fourier transform (IFT) approach, which determines a histogram of all distances within the scatterer.[6] The function is parametrized by the input parameters of the maximum dimension of the scatterer (D_{max}) and a regularization constant (α). From the p(r) the radius of gyration of the scatterer (R_g) and the extrapolated forward intensity (*I(0)*) were calculated using the following equations:

$${R_g}^2 = \frac{\int_0^{D_{max}} p(r) r^2 dr}{2\int_0^{D_{max}} p(r) dr}$$

$$I(0) = 4\pi \int_0^{D_{max}} p(r) dr$$

To obtain information on the collective behavior of the protein, the aggregation number (N_{agg}) was calculated from the scattering data as follows:

$$N_{agg} = \frac{V}{V_m}$$

$$V = \sqrt{\frac{I(0)\, M_w}{c\, \Delta SLD^2\, N_A}}$$

where *V* is the experimental volume of the scatterer, V_m is the volume of the protein monomer, *c* is the concentration of protein in the sample (in g cm^{-3}), *ΔSLD* is the scattering excess (i.e. the difference between the scattering length density of the protein and that of the solvent, SLD_p-SLD_s), and *NA* is Avogadro's number. The scattering length density and monomer volume of the

proteins were calculated using the sequence of each protein (PDB files: BSA – 4F5S; Lyz – 1HEW) and the Biomolecular Scattering Length Density Calculator from the Science and Technology Facilities Council, UK.[7] The SLDs of the solvents are presented in Table S1:

Table S1 Scattering length density of the solvents used in the SANS characterization.

Solvent	SLD / $\times 10^{-6}$ Å^{-2}
1:2 choline-d_9 chloride:glycerol-d_8	6.40
1:2 choline-d_9 acetate-d_3:glycerol-d_8	6.32
1:2 choline-d_9 chloride:urea-d_4	6.05
1:2 choline-d_9 chloride:acetic acid-d_4	6.41
D_2O	6.37

The calculated p(r)'s were modelled using two gaussian functions to determine the position and height of the peaks for Lyz and BSA, where the position of the first peak (r_1) constitutes a descriptive parameter related to the folding state of the protein monomer.

In addition, the modelling of the protein scattering was performed using a generalized form of the Porod approximation.[8] This model determines the Porod exponent *m* (or *n*) using the following equation:

$$\log\big(I(q)\big) = log(A) - m\ log(q)$$

The parameter *m* relates to the fractal dimension of the scatterer and for polymer coils can adopt different values depending on the polymer-polymer and solvent-polymer interactions. As such, a value for the slope of 3 relates to a collapsed polymer coil, 2 is a signature of Gaussian chains and 5/3 is for fully swollen polymer coils.[9]

The analysis using the IFT method was performed in the ATSAS 3.2.1 suite.[10] The analysis of data using the generalized Porod approximation was performed on SasView 5.0.3.[11]

2 Results from the characterization of the proteins

Table S2 Parameters derived from the analysis of the spectroscopy data for Lyz and BSA in different DESs and aqueous buffer presented in Figure 2 and 4: $\lambda_{d2,Tyr}$ – Tyr peak position obtained from the peak analysis of the second-derivative UV-vis spectra, $\lambda_{max,Trp}$ – position of the emission maximum for the Trp peak, and $[\theta]_{MR}$ – mean residue ellipticity at 222 nm.

Solvent	$\lambda_{d2Abs,Tyr}$ / nm	$\lambda_{max,Trp}$ / nm	$[\theta]_{MR}$ @222 nm / 10^{-3} mdeg cm^2 dmol^{-1}
Lyz			
1:2 ChCl:Glyc	288.7±0.1	346±1	-11135±621
1:2 ChAcO:Glyc	288.9±0.3	-	-6152±338
1:2 ChCl:Urea	288.3±0.2	345±1	-10871±572
1:2 ChCl:AcOH	287.9±0.1	337±1	-1331±64
Aqueous buffer	288.2±0.2	347±1	-11317±710
BSA			
1:2 ChCl:Glyc	283.6±0.4	342±2	-17500±784
1:2 ChAcO:Glyc	284.9±0.5	-	-11733±492
1:2 ChCl:Urea	283.3±0.7	342±1	-590±51
1:2 ChCl:AcOH	283.0±0.8	338±1	-145±27
Aqueous buffer	282.4±0.3	354±1	-21892±894

Table S3 Parameters derived from the analysis of the SANS data presented in Figure 3 and 5: D_{max} – maximum dimension of the scatterer, r_1 – the apparent size of the protein monomer parameterized as the position of the first peak in the p(r), N_{agg} – the average number of protein units in a self-association equilibrium, and m and n – negative Porod slopes at high and low q, respectively.

Solvent	D_{max} / Å	r_1 / Å	N_{agg}	m	n
Lys					
1:2 ChCl:Glyc	41.7	13.3±0.2	1.08±0.06	4.4±0.3	0.11±0.08
1:2 ChAcO:Glyc	40.8	14.0±0.3	1.22±0.05	3.9±0.3	0.18±0.11
1:2 ChCl:Urea	41.5	14.9±0.5	0.92±0.14	4.3±0.4	0.17±0.15
1:2 ChCl:AcOH	>550	78±6	>>12	2.5±0.2	1.7±0.2
Aq. buffer	39.8	12.9±0.1	1.08±0.04	4.2±0.2	0.18±0.07
BSA					
1:2 ChCl:Glyc	278	57.8±1.6	1.68±0.10	3.5±0.4	0.02±0.04
1:2 ChAcO:Glyc	283	60.3±1.1	2.30±0.24	3.6±0.6	0.23±0.13
1:2 ChCl:Urea	>550	>200	>>6	1.2±0.4	3.0±0.1
1:2 ChCl:AcOH	>550	>200	>>6	1.6±0.2	2.9±0.1
Aq. buffer	138	34.5±0.6	1.37±0.05	4.0±0.2	0.24±0.11

3 Validation of the exponential component analysis from excited-state emission fluorescence

Initially, we aimed to determine the most suitable approach to analyze our data by using an increasing number of decay components, i.e., one, two, and three. As expected, the addition of extra components to the theoretical model increased the

quality of the fit (Figure S4 and Table S4). However, beyond two components, the gains were only marginal.

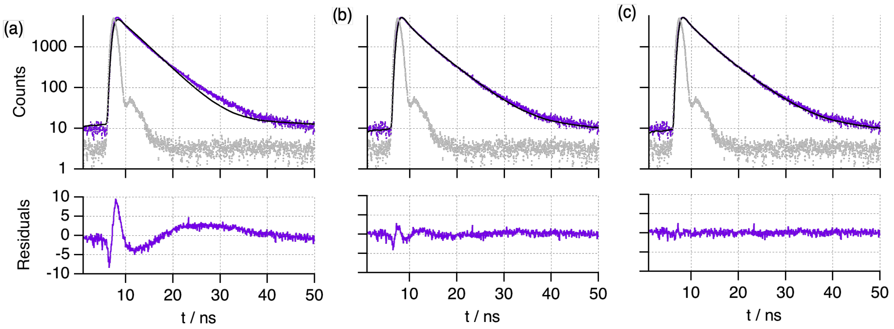

Figure S4 Excited-state emission fluorescence data, fits, and residuals of Lyz in 1:1:1 ChCl:Glyc:AcOH using a different number of components: (a) one, (b) two, and (c) three. The instrument response function (IRF) is presented in each plot as grey dots.

Table S4 Parameters derived from the exponential component analysis presented in Figure S4.

Number of components	$<\tau>_{int}$ / ns	χ^2
1	3.92	6.19
2	3.97	1.33
3	3.91	1.10

4 Analysis from the two-state denaturation model

To probe the possibility of co-existing conformations, e.g., variable populations of folded and unfolded states,[12] data were analyzed using a two-state denaturation model. This approach uses four characteristic lifetimes, two associated to the folded protein (τ_{F1} and τ_{F2}) and two to the unfolded state (τ_{U1} and τ_{U2}) with variable populations of each component:

$$I(t) = B + \alpha_{F1} e^{\frac{t}{\tau_{F1}}} + \alpha_{F2} e^{\frac{t}{\tau_{F2}}} + \alpha_{U1} e^{\frac{t}{\tau_{U1}}} + \alpha_{U2} e^{\frac{t}{\tau_{U2}}}$$

This approach was used to analyze the data for Lyz in 1:1:1 ChCl:Glyc:AcOH. The results from the fit are presented in Figure S5 and Table S5.

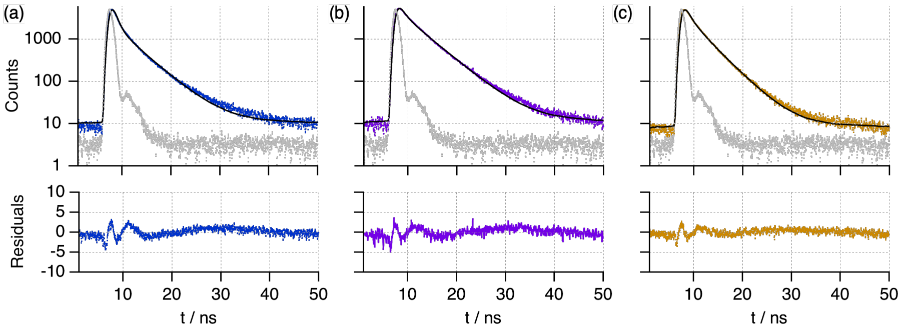

Figure S5 Excited-state emission fluorescence data, fits, and residuals of Lyz in (a) 1:2:0, (b) 1:1:1, and (c) 1:0:2 ChCl:Glyc:AcOH using a two-state denaturation model. The instrument response function (IRF) is presented in each plot as grey dots.

Table S5 Parameters derived from the analysis using a two-state denaturation model presented in Figure S5.

System	α_{F1}	τ_{F1} / ns	α_{U1}	τ_{U1} / ns	α_{F2}	τ_{F2} / ns	α_{U2}	τ_{U2} / ns	χ^2
1:2:0 ChCl:Glyc:AcOH	0.0319	0.869	0	1.21	0.0080	3.81	0	4.37	1.22
1:1:1 ChCl:Glyc:AcOH	-0.0036	0.869	0.0195	1.21	-0.0086	3.81	0.0250	4.37	1.52
1:0:2 ChCl:Glyc:AcOH	0	0.869	0.0191	1.21	0	3.81	0.0132	4.37	1.34

This approach to fit the data was found unsuccessful. Besides we observed an increase in the χ^2 parameter compared to the floating double exponential model (from 1.33 to 1.52), the two-state denaturation model yielded negative α_i values for some components, thus resulting physically unrealistic. As such, we concluded that the variation in the composition of the ternary DESs leads to the stabilization of discrete conformational intermediates instead of co-existing folded and unfolded proteins.

5 Excited-state emission fluorescence of lysozyme in aqueous buffer

To validate our analysis protocol, we determined the excited-state emission fluorescence for Lyz in aqueous buffer and compared the results to literature values. The results from the characterization are presented in Figure S6.

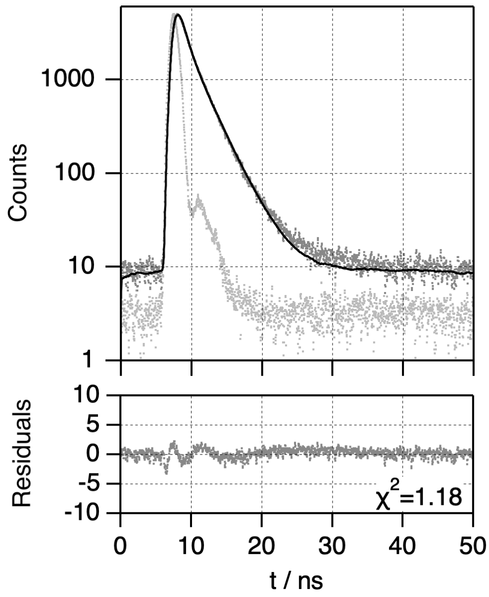

Figure S6 Excited-state emission of Lyz in 10 mM, pH 7 phosphate buffer at 25 °C. The solid line represents the fit using a double exponential decay function calculated using the reconvolution method. The IRF is shown as light grey dots. Residuals and the χ2 parameter from the fit are provided in the bottom panel.

The control results show two distinct lifetimes for Lyz in aqueous buffer, a fast component <1 ns and a slow component at 2.6 ns. This agrees with previous reports on the characteristic decay of the excitation of the aromatic residues of the protein.[4, 13]

6 Densities and viscosities of the DESs

Table S6 Densities and viscosities of the ternary DESs and their binary analogues. The error bars correspond to the standard deviation from three repeats.

Solvent	ρ / g cm^{-3}	ν / m2 s^{-1}	η / mPa s
1:2:0 ChCl:Glyc:AcOH	1.1908	268.7±0.3	320.0±0.4
1:1.9:0.1 ChCl:Glyc:AcOH	1.1859	231.1±0.7	274.0±0.8
1:1.8:0.2 ChCl:Glyc:AcOH	1.1815	225.8±1.1	266.8±1.3
1:1.7:0.3 ChCl:Glyc:AcOH	1.1771	203.8±1.9	239.9±2.2
1:1.5:0.5 ChCl:Glyc:AcOH	1.1683	184.7±0.4	215.8±0.5
1:1:1 ChCl:Glyc:AcOH	1.1458	114.2±3.1	130.9±3.6
1:0.5:1.5 ChCl:Glyc:AcOH	1.1246	74.93±0.19	84.27±0.22
1:0:2 ChCl:Glyc:AcOH	1.1038	43.9±0.61	48.46±0.67

7 References

1. Colomines, G.; Decaen, P.; Lourdin, D.; Leroy, E., Biofriendly ionic liquids for starch plasticization: a screening approach. *RSC Advances* **2016,** *6* (93), 90331-90337.

2. Sanchez-Fernandez, A.; Basic, M.; Xiang, J.; Prevost, S.; Jackson, A. J.; Dicko, C., Hydration in Deep Eutectic Solvents Induces Non-monotonic Changes in the Conformation and Stability of Proteins. *J. Am. Chem. Soc.* **2022,** *144* (51), 23657-23667.

3. Dewhurst, C. D., Graphical reduction and analysis small-angle neutron scattering program: GRASP. *J. Appl. Crystallogr.* **2023,** *56* (Pt 5), 1595-1609.

4. Alcala, J. R.; Gratton, E.; Prendergast, F. G., Fluorescence lifetime distributions in proteins. *Biophys. J.* **1987,** *51* (4), 597-604.

5. Hosny, N. A.; Mohamedi, G.; Rademeyer, P.; Owen, J.; Wu, Y.; Tang, M.-X.; Eckersley, R. J.; Stride, E.; Kuimova, M. K., Mapping microbubble viscosity using fluorescence lifetime imaging of molecular rotors. *Proceedings of the National Academy of Sciences* **2013,** *110* (23), 9225-9230.

6. Glatter, O., A new method for the evaluation of small-angle scattering data. *J. Appl. Crystallogr.* **1977,** *10* (5), 415-421.

7. Clifton, L. A.; Myatt, D. Protein Scattering Length Density Calculator v1.0. http://psldc.isis.rl.ac.uk/Psldc/.

8. Hammouda, B., A new Guinier–Porod model. *J. Appl. Crystallogr.* **2010,** *43* (4), 716-719.

9. Hammouda, B., *Probing nanoscale structures - The SANS toolbox*. 2008.

10. Manalastas-Cantos, K.; Konarev, P. V.; Hajizadeh, N. R.; Kikhney, A. G.; Petoukhov, M. V.; Molodenskiy, D. S.; Panjkovich, A.; Mertens, H. D. T.; Gruzinov, A.; Borges, C.; Jeffries, C. M.; Svergun, D. I.; Franke, D., ATSAS 3.0: expanded functionality and new tools for small-angle scattering data analysis. *J. Appl. Crystallogr.* **2021,** *54* (Pt 1), 343-355.

11. Doucet, M.; Cho, J. H.; Alina, G.; Attala, Z.; Bakker, J.; Bouwman, W.; Butler, P.; Campbell, K.; Cooper-Benun, T.; Durniak, C.; Forster, L.; Gonzales, M.; Heenan, R.; Jackson, A.; King, S.; Kienzle, P.; Krzywon, J.; Nielsen, T.; O'Driscoll, L.; Potrzebowski, W.; Prescott, S.; Ferraz Leal, R.; Rozycko, P.; Snow, T.; Washington, A. SasView version 5.0.3. https://zenodo.org/record/3930098 (accessed 2020-08-24).

12. Gratton, E.; Silva, N.; Mei, G.; Rosato, N.; Savini, I.; Finazzi-Agro, A., Fluorescence lifetime distribution of folded and unfolded proteins. *International Journal of Quantum Chemistry* **1992,** *42* (5), 1479-1489.

13. Rmoso, C.; Forster, L. S., Tryptophan fluorescence lifetimes in lysozyme. *J. Biol. Chem.* **1975,** *250* (10), 3738-3745.
